# Shifting patterns of mite sensitization in Spain and Portugal: real-world diagnostic and immunotherapy practices (MITE-WISE project)

**DOI:** 10.3389/falgy.2026.1946434

**Published:** 2026-10-28

**Authors:** Ruperto González-Pérez, Mónica Antón, Manuel J. Rial, Javier Figueroa

**Affiliations:** 1 Allergy Section, Hospital Universitario de Canarias, Santa Cruz de Tenerife, Spain; 2 Allergy Section, Hospital Universitario San Juan de Alicante, Alicante, Spain; 3 Allergy Section, Complexo Hospitalario Universitario de Ferrol, A Coruña, Spain; 4 Allergy Section, Hospital Universitario Insular de Gran Canaria, Instituto de Investigación Sanitaria de Canarias (IISC), Las Palmas, Spain

**Keywords:** allergen immunotherapy, *Blomia tropicalis*, cosensitization, *Dermatophagoides*, house dust mite, *Lepidoglyphus destructor*, real-world evidence

## Abstract

**Introduction:**

Mite allergy sensitization profiles in Southern Europe are evolving. While *Dermatophagoides spp*. remain the most prevalent allergens associated with allergic rhinitis, asthma and atopic dermatitis, sensitization to *Lepidoglyphus destructor* and *Blomia tropicalis* has been increasingly reported in Iberian regions. Diagnostic and allergen immunotherapy (AIT) strategies may not reflect this shift.

**Methods:**

Real-world, mixed-methods study guided by a Scientific Committee (SC) of allergists, comprising an online questionnaire completed by 215 allergists and semi-structured interviews with an Extended SC.

**Results:**

Most specialists managed patients sensitized to *Dermatophagoides spp.* and *L. destructor* (97.7%) and to *Dermatophagoides spp.* and *B. tropicalis* (70.7%), reflecting allergist-reported clinical experience with these emerging mites. The eight interviewed experts perceived an increasing recognition of sensitization to *L. destructor* and *B. tropicalis*, particularly of *B. tropicalis* in the Canary Islands and along the Portuguese coast. Despite this, reported testing practices differed: 85.2% (179/210) reported always testing *L. destructor*, whereas 36.8% (56/152) reported routinely testing *B. tropicalis*, a descriptive difference that may reflect heterogeneous diagnostic practices. Among specialists who reported always testing the respective mite, 98.9% (177/179) of those testing *L. destructor* reported prescribing AIT targeting *Dermatophagoides spp.* and *L. destructor*, and 91.1% (51/56) of those testing B. tropicalis reported prescribing AIT targeting *Dermatophagoides spp*. and *B. tropicalis*, in patients with reported clinically relevant sensitization. All respondents reported a preference for subcutaneous AIT, and 98% of AIT prescribers reported using polymerized extract mixtures.

**Conclusion:**

Among participating allergists, reported clinically relevant sensitization to *Dermatophagoides spp*. together with *L. destructor* or *B. tropicalis* was frequently followed by reported subcutaneous AIT within the subgroups that routinely tested the respective mite. Given the recruitment of AIT-experienced allergists, these proportions should not be generalized to all specialists. Testing was reported less frequently for *B. tropicalis* than for *L. destructor*. Whether region-specific inclusion of these mites in diagnostic protocols would improve diagnosis or treatment outcomes remains to be established in studies with objective confirmation of clinical relevance.

## Introduction

Mites are a major source of indoor allergens associated with asthma, allergic rhinitis and atopic dermatitis (1, 2). In Europe, *Dermatophagoides pteronyssinus* and *Dermatophagoides farinae* (*Dermatophagoides* spp.) are the predominant mite species (1). However, climate change, increasing temperature and humidity, population mobility and urbanization are reshaping allergen distribution and exposure (3–5). These environmental changes are particularly relevant for mites, whose prevalence and geographic distribution are strongly influenced by local conditions. Consequently, mite species historically considered less prevalent are being detected more frequently in some areas (3–5), and environmental data suggest increasing exposure to both classical and non-pyroglyphid mites (6, 7).

Sensitization profiles are heterogeneous, and polysensitization is frequent, with geographical variation in molecular patterns that can complicate diagnosis and treatment (6). In Spain and Portugal, *Blomia tropicalis* (*B. tropicalis*) and *Lepidoglyphus destructor* (*L. destructor*) are increasingly reported (3–5). Both species have been identified in more than 30% of domestic dust samples in Spain (3). In the Canary Islands, *B. tropicalis* sensitization has been reported in approximately 21% of young adults and associated with asthma, bronchial hyperresponsiveness, rhinitis and dermatitis (8). These findings support the clinical relevance of non-pyroglyphid mites in specific climatic and microenvironmental settings (1, 4).

Despite this evolving epidemiology, diagnostic strategies may not fully reflect current sensitization patterns. EAACI environmental guidance does not comprehensively address *B. tropicalis* or *L. destructor*, leaving a gap in regions where these mites may be clinically relevant but are not routinely integrated into diagnostic algorithms or AIT recommendations (1, 2, 5, 9, 10). This mismatch between real-world exposure and diagnostic evaluation may lead to under-recognition of relevant sensitizations. Importantly, this gap does not appear to extend equally to treatment. According to EAACI and other major allergy guidelines, AIT is a disease-modifying treatment for IgE-mediated respiratory allergy and is an established add-on option for mite-driven allergic asthma and rhinitis (2, 11, 12). In Spain, allergen extract mixtures are commonly used in polysensitized patients when each component has demonstrated clinical relevance (3, 4, 13), an approach also supported by the Spanish REAL project (14).

There is a need to clarify to what extent *B. tropicalis* and *L. destructor* are considered in the diagnostic workup of mite-allergic patients and to what degree they are included in AIT formulations when sensitization is present. In this context, the MITE-WISE project was designed to describe how diagnostic and therapeutic practices for patients with clinically relevant sensitization to *Dermatophagoides spp.* and *L. destructor* or *B. tropicalis* are adapting to the current epidemiological situation observed in Spain and Portugal, within the context of evolving environmental exposure and sensitization profiles, and to identify potential gaps and areas for improvement in diagnostic screening and the use of specific AIT.

## Methods

### Study design

MITE-WISE was a mixed-methods project comprising two complementary components: an anonymized online questionnaire administered to allergy specialists with experience in AIT across Spain and Portugal, in which identifying information was collected for registration purposes and subsequently separated from the analytical dataset, and semi-structured online interviews with an extended expert Scientific Committee (SC) of allergists, designed to provide in-depth, region-specific interpretation of the questionnaire findings. The qualitative component was intended primarily to contextualize and support the interpretation of the questionnaire findings, rather than to constitute an independent analytical dataset or a formal triangulation procedure. Where expert perspectives diverged from, or nuanced, the quantitative results, such discrepancies were reported descriptively as regional variations in perception rather than reconciled through a formal integration framework.

### Participants’ recruitment

The present study employed a closed, email-based survey using a non-probability convenience sampling approach. Potential participants were identified from a contact database of allergists practising in Spain and Portugal that was maintained by Adelphi Targis on behalf of LETI Pharma. The database was used as the sampling frame to identify contactable clinicians and was not designed to be a representative registry of all allergy specialists in either country. Eligible participants were allergists practising in Spain or Portugal who reported routinely managing patients with dust mite allergy and previous experience prescribing AIT. These eligibility criteria were intended to identify clinicians with relevant experience; they did not make the sample representative of all allergists in Spain and Portugal. Recruitment took place between 16 June and 15 July 2025. A total of 215 unique clinicians were invited; all consented, completed the questionnaire and met the eligibility criteria, resulting in a 100% participation and completion rate. No non-responders were recorded. Invitations were distributed only after eligibility had been pre-confirmed during the identification of potential participants from the database, which accounts for the complete participation observed; no invitations were returned undelivered, declined or excluded after distribution. No formal *a priori* sample-size calculation was performed because the study was exploratory and descriptive. Identifying registration data were separated from the analytical dataset before analysis, and all participants provided informed consent.

### Questionnaire development and structure

The questionnaire and interview guide were developed by a SC of four Spanish allergists with extensive experience in mite allergy and AIT. The online questionnaire, designed to be completed in approximately 20 min, comprised 96 questions with the following sections: characteristics and description of participant profiles; detection and diagnosis of dust mite allergy; and aetiological treatment with AIT in patients allergic to mites. The questionnaire was developed by the SC through iterative review and consensus refinement to ensure clarity and feasibility. For the present manuscript, analyses were restricted to questionnaire items directly addressing the predefined study objectives related to the diagnostic assessment and AIT management of *B. tropicalis* and *L. destructor*. The complete questionnaire is provided as Supplementary Material to support reproducibility. Throughout the questionnaire and the present manuscript, clinically relevant sensitization refers to sensitization considered clinically relevant by the responding allergist (reported clinically relevant sensitization); the criteria and diagnostic procedures used by respondents to establish clinical relevance were not collected, and clinical relevance was not independently validated in this study. Before field implementation, the questionnaire underwent internal pre-testing for wording, structure, functionality and feasibility, followed by iterative expert review and consensus refinement by the Scientific Committee; no formal external pilot study was performed.

### Qualitative interviews

The Extended SC comprised 8 allergists selected to represent different regions across Spain and Portugal, with longstanding experience in mite allergy and AIT in their respective geographic areas. Experts were purposively selected by the SC and were required to fulfil the same strict, predefined eligibility criteria applied to study participants: being specialists in Allergy, routinely managing patients with dust mite allergy, practicing in Spain or Portugal, and having previous experience prescribing AIT. This selection aimed to maximise regional and practice-pattern diversity rather than statistical representativeness. Interviews were conducted by members of the coordinating team online, recorded and fully transcribed. Transcripts were reviewed by the coordinating team and summarised narratively around the predefined topics of the semi-structured interview guide; no formal qualitative coding framework, independent double coding by multiple analysts, or software-assisted thematic analysis was applied. Accordingly, the qualitative findings presented in this manuscript should be interpreted as expert contextualization of the questionnaire results rather than as a formally analysed qualitative dataset, and points of divergence among experts were reported as regional perspectives rather than resolved through consensus procedures. The complete semi-structured interview guide is provided as Supplementary Material to support reproducibility.

### Statistical analyses

Quantitative data were analysed descriptively using absolute and relative frequencies for categorical variables and numerical descriptive measures such as means or medians, as appropriate. For key outcomes, results were calculated both on the total sample and on the relevant denominators. Due to unequal sample sizes per region and the exploratory nature of the project, no formal hypothesis testing between regions was performed. Accordingly, geographical differences are described qualitatively and should not be interpreted as statistically demonstrated.

### Ethics statement

This study involved human participants, namely 215 allergists who completed the online questionnaire and 8 allergists who participated in the semi-structured interviews; no patients were involved and no patient-level data were collected. Invitations containing a unique questionnaire link were distributed by email. Participation was voluntary, anonymized, and experts received economic compensation in accordance with standard practices for questionnaire-based research and consistent with the time spent participating. Given the descriptive and practice-oriented nature of the project, and the fact that no patient-level data were collected, no formal ethics committee review was sought and no formal institutional determination of exemption was requested; this assessment was made by the study team on the basis of Spanish biomedical research regulations (Ley 14/2007) (15). All participating clinicians provided informed acceptance to use their anonymized answers for research and publication purposes. The study involved exclusively the voluntary participation of consenting healthcare professionals reporting on their professional practice, and interview recordings and transcripts were handled confidentially and anonymized for analysis.

## Results

### Specialists’ profile

A total of 215 clinicians (100% allergists) completed the questionnaire (Spain, *n* = 194; Portugal, *n* = 21). Of these, 116 (54%) reported more than 15 years of experience in allergy, and all had previous experience in prescribing AIT, with 114 (53%) having used AIT for over 15 years. On average, respondents carried out 77.5% of their clinical practice in public hospitals, with broad geographic spread across Spanish autonomous communities and Portuguese regions (Figure 1).

**Figure 1:**
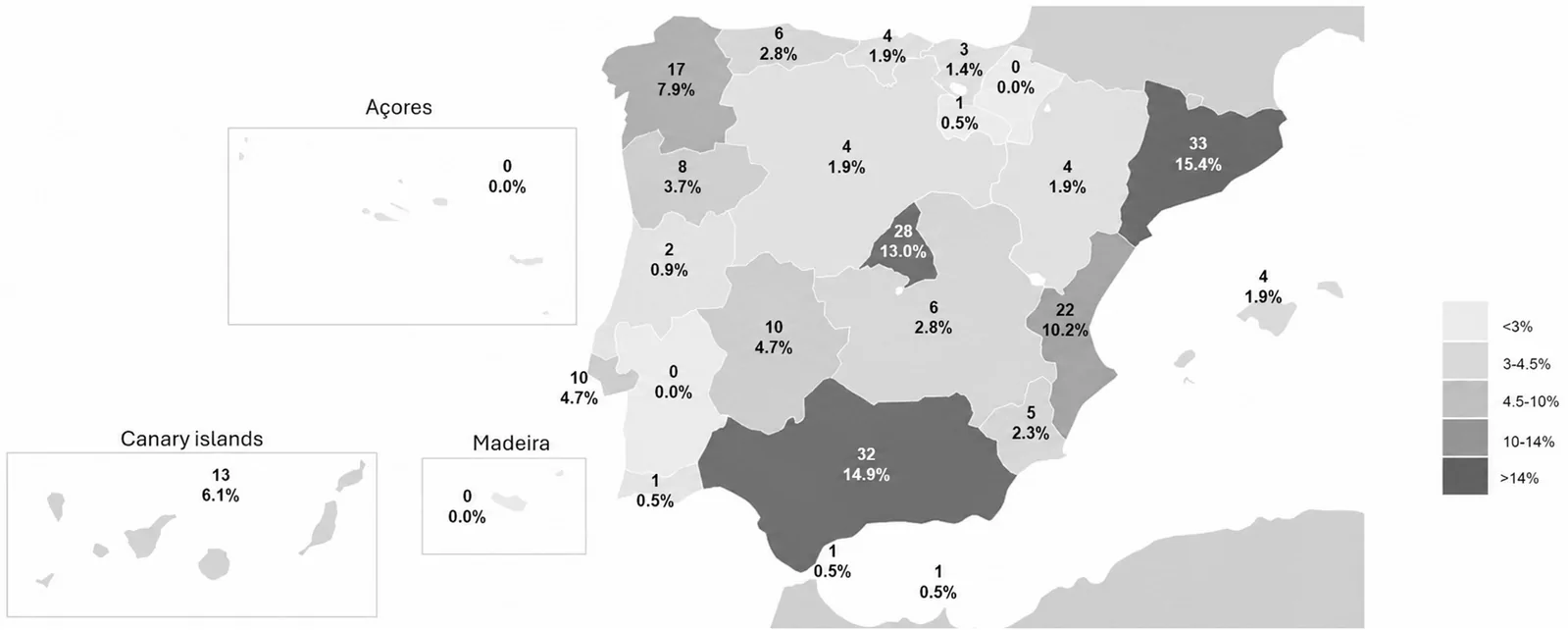
Geographical distribution of allergy specialists (*n*/%) who participated in the questionnaire.

### Mite sensitization in clinical practice

All respondents managed patients allergic to *Dermatophagoides spp.* in their routine practice. A total of 70.7% (152/215) of respondents reported managing patients with reported clinically relevant sensitization to *Dermatophagoides spp.* and *B. tropicalis*, and 97.7% (210/215) reported managing patients with reported clinically relevant sensitization to *Dermatophagoides spp.* and *L. destructor* (Figure 2).

**Figure 2:**
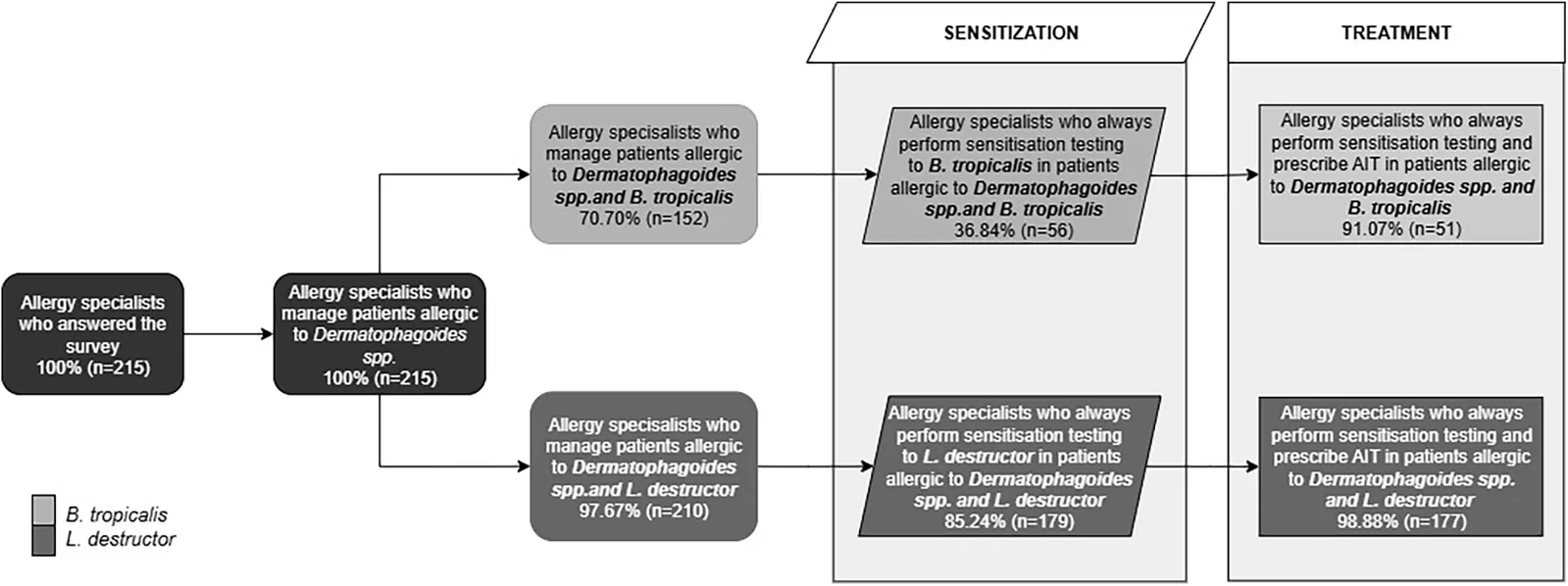
Clinical practice algorithm of allergy specialists who manage patients allergic to *Dermatophagoides spp*. and B. tropicalis or L. destructor. Percentages have been calculated using the denominator shown in each preceding group.

### Diagnostic approach

Among clinicians managing patients with reported clinically relevant sensitization to *Dermatophagoides spp.* and *B. tropicalis* (*n* = 152), 36.8% (56/152) stated that they always performed sensitization testing to *B. tropicalis* in patients with *Dermatophagoides spp.* allergy. In contrast, among those managing patients with reported clinically relevant sensitization to *Dermatophagoides spp.* and *L. destructor* (*n* = 210), 85.2% (179/210) reported always including *L. destructor* in their sensitization diagnostic screening panel. Although percentages varied across regions, these differences are presented descriptively; overall, routine testing was reported more frequently for *L. destructor* than for *B. tropicalis* (Figure 3).

**Figure 3:**
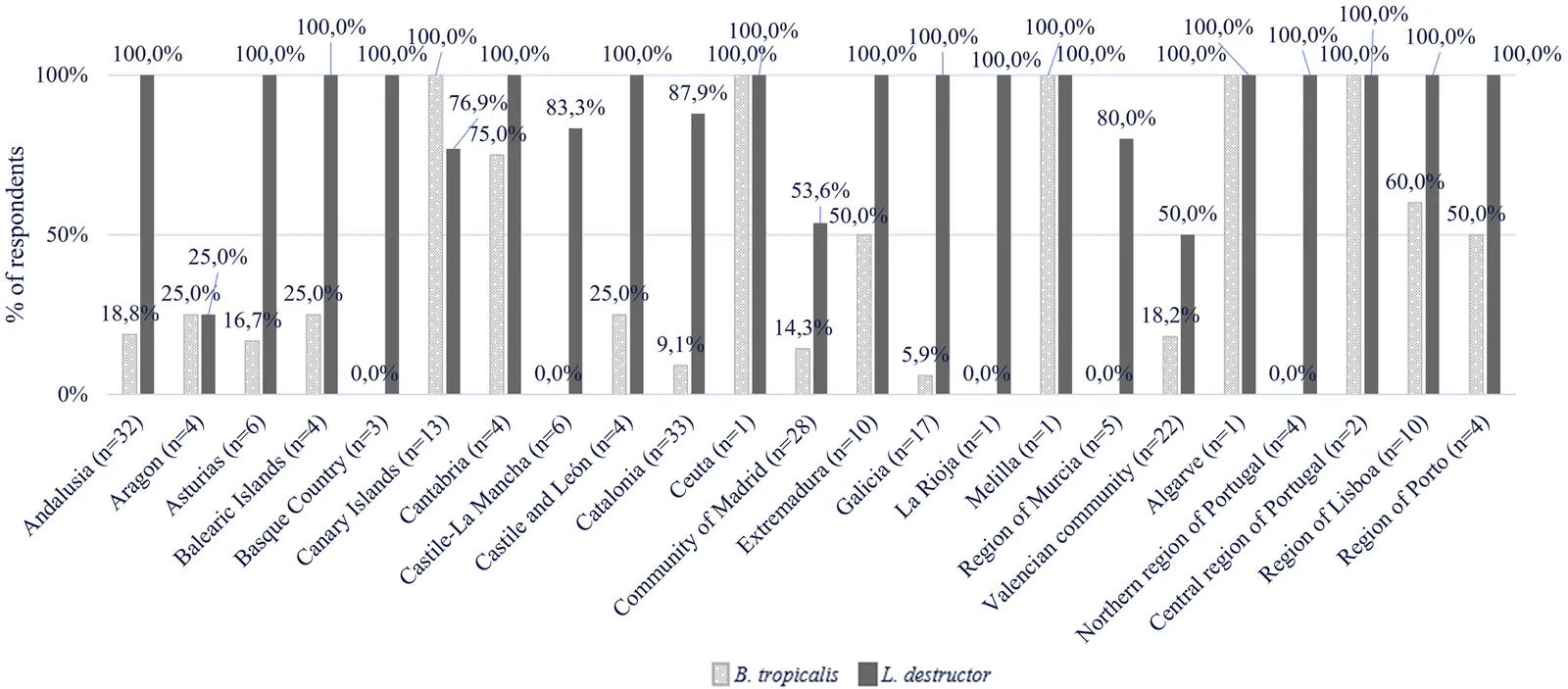
Geographical distribution of allergy specialists who always perform sensitization testing to *B. tropicalis or L. destructor* in patients allergic to *Dermatophagoides spp.* Response to the question: In patients with allergy to *Dermatophagoides spp.,* do you always perform a sensitization study for *B. tropicalis or L. destructor*?

### Determinants and barriers to diagnostic testing

A detailed clinical history was the diagnostic procedure of preference among specialists (mean 8.6/10 on a scale of 1–10, where 1 = ‘not relevant at all’; 10 = ‘extremely relevant’), followed by skin prick testing (mean 8.4/10) and *in vitro* tests, including complete allergen extract or component-resolved diagnostics (mean 6.5/10). Among specialists who routinely tested *B. tropicalis* or *L. destructor* alongside *Dermatophagoides spp.* in patients evaluated for mite allergy, the determinants with the highest mean scores for encouraging sensitization testing were the patient's geographic zone (mean 8.3/10), either place of origin or residence considering its temperature and humidity; previous AIT failure (mean 7.8/10); and insufficient symptom control despite standard management (mean 7.6/10). Clinicians also identified several barriers to optimal diagnostic characterization: the lack of updated acarological maps reflecting current distributions of *B. tropicalis* and *L. destructor* (mean 7.5/10), the absence or limited availability of data reflecting sensitization profiles (mean 7.3/10), and uncertainty regarding the clinical relevance in a specific area (mean 7.0/10).

### Allergen immunotherapy practices

Among specialists who routinely tested *B. tropicalis* alongside *Dermatophagoides spp.* in patients evaluated for mite allergy, 91.1% (51/56) reported prescribing AIT in patients with reported clinically relevant sensitization to both mites. Among those who always tested for *L. destructor*, 98.9% (177/179) prescribed AIT in patients with reported clinically relevant sensitization to these mites (Figure 4). The main reasons cited for prescribing AIT in these patients were the need to improve quality of life (mean 8.7/10), the severity of symptoms (mean 8.7/10) and symptom control (mean 8.2/10). All respondents (215/215) reported subcutaneous administration as their preferred route of AIT. Furthermore, 98% of specialists who reported prescribing AIT targeting *Dermatophagoides spp.* and *B. tropicalis* or *L. destructor* reported using polymerized extract mixtures in patients with reported clinically relevant sensitization to both mites. These proportions represent reported prescribing practices among participating specialists rather than objectively measured prescription patterns. Because eligibility required previous experience prescribing AIT, these proportions describe AIT-experienced allergists and should not be extrapolated to all allergy specialists.

**Figure 4:**
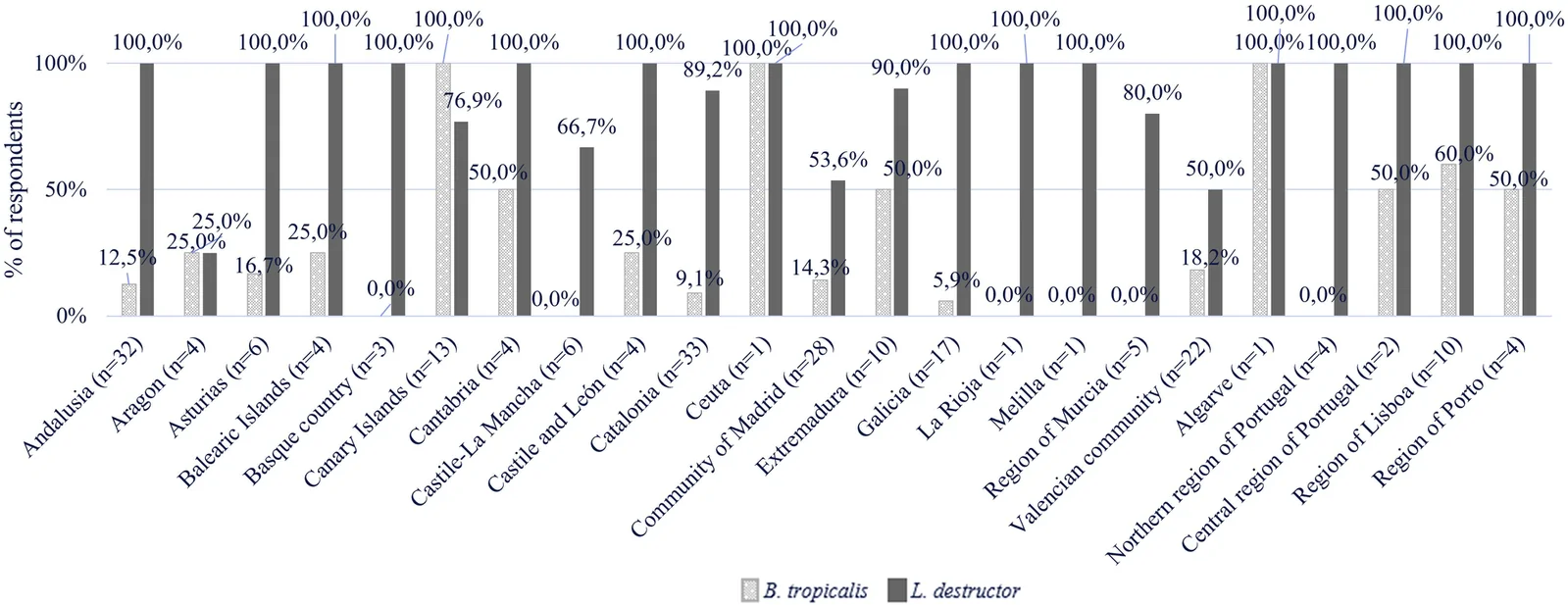
Geographical distribution of allergy specialists who always perform sensitization testing and prescribe AIT to patients allergic to *Dermatophagoides spp. and B. tropicalis or L. destructor*. Response to the question: Thinking about the total of your current professional activity, approximately: in what percentage of the patients you see on average each month who have a diagnosis of allergy to *Dermatophagoides spp.* and *B. tropicalis/ L. destructor* do you recommend aetiological treatment with AIT against both mites?

### Barriers to allergen immunotherapy implementation

The main barriers identified for the optimal use of AIT targeting *Dermatophagoides spp.* and *B. tropicalis* or *L. destructor* were the perception of limited robust evidence (mean 7.8/10), the lack of knowledge regarding the characterization and standardization of allergenic extracts to ensure stability, safety and efficacy (mean 7.7/10), and the absence of clear clinical guidelines or protocols for the management of these allergies (mean 7.7/10).

### Qualitative interview findings

Qualitative interviews with the eight Extended SC allergists were consistent with the questionnaire findings and highlighted the influence of geographical and environmental context on diagnostic practice. Given the small number of interviewees and the unequal representation of regions, these observations reflect individual expert perceptions and should not be interpreted as quantified regional differences. Coastal and humid regions, including the Portuguese coast, the Balearic Islands, Andalusia and Catalonia, were perceived as areas where *B. tropicalis* and *L. destructor* had greater clinical relevance and were more frequently considered in testing. In other mainland regions, particularly for *B. tropicalis*, testing was reported to be more selective and guided by clinical suspicion or component-resolved diagnostics. The Canary Islands were identified as a distinct setting, with more consistent incorporation of *B. tropicalis* into routine testing. Across regions, *L. destructor* was perceived as more widely integrated into clinical practice than *B. tropicalis*, in line with the quantitative results.

## Discussion

MITE-WISE suggests that, as reported by participating allergists, routine diagnostic testing for non-*Dermatophagoides* mites is unevenly implemented, with *L. destructor* included in screening panels more frequently than *B. tropicalis*. Testing was mainly influenced by geographical context, previous AIT failure and insufficient symptom control, whereas key barriers were the lack of updated acarological maps, limited sensitization data and uncertainty regarding local clinical relevance. When clinically relevant sensitization was reported, most specialists within the subgroups that routinely tested the respective mite reported prescribing AIT, predominantly by the subcutaneous route. Qualitative interviews supported these patterns and the influence of geographical and environmental factors on clinical decision-making.

The observation that nearly all respondents managed patients with reported clinically relevant sensitization to *Dermatophagoides spp.* and *L. destructor*, and that a substantial proportion reported managing patients with clinically relevant sensitization to *Dermatophagoides spp.* and *B. tropicalis*, is consistent with previous European and Iberian studies documenting frequent sensitization between pyroglyphid and non-pyroglyphid mites and demonstrating that *L. destructor* sensitization is linked to more severe asthma and perennial rhinitis, particularly in humid and coastal environments (4, 5, 13, 16, 17). Sensitization to storage mites has been reported in different European populations, although estimates vary substantially according to geographic setting, population selection, mite species and diagnostic method (16). These reports are consistent with clinicians’ perception of the clinical relevance of both species and with environmental surveys documenting their presence in over 30% of domestic dust samples in Spain (3). However, the observed disparity between the proportion of specialists who routinely tested for *L. destructor* compared with *B. tropicalis* suggests a diagnostic asymmetry. Since the relative clinical significance of both mites was not assessed in this study, this difference cannot be attributed to under-recognition alone and may reflect differences in familiarity, availability of diagnostic tools, regional epidemiology, or the degree of integration of *B. tropicalis* into standard screening protocols, particularly in regions where its presence has only recently been recognized.

The main determinants encouraging specialists to perform sensitization testing for these emerging mites were the patient's geographic zone of residence or origin, previous AIT failure, and insufficient symptom control despite standard management. These findings indicate that diagnostic decisions are closely tied to clinical and environmental context. At the same time, the principal barriers identified (absence of updated acarological maps, limited availability of regional sensitization data, and uncertainty regarding local clinical relevance) suggest that, from the respondents’ perspective, limitations in the information available to clinicians may contribute to this pattern more than a lack of clinical awareness. Qualitative interview findings were consistent with this interpretation and provided additional, perception-based context: interviewed experts from coastal and humid regions, including the Portuguese coastline, the Balearic Islands, Andalusia, and Catalonia, described higher perceived relevance and more consistent testing for *B. tropicalis* and *L. destructor*, whereas specialists in other areas of mainland Spain reported more selective or non-systematic approaches, often relying on component-resolved diagnostics or specific clinical suspicion. The interviewed experts described the Canary Islands as a distinct setting, where longstanding exposure to *B. tropicalis* was considered to have led to its routine inclusion in standard testing panels. Across all regions, *L. destructor* was perceived as more widely integrated into clinical practice than *B. tropicalis*, which aligns with the quantitative asymmetry in diagnostic testing rates. Interviewed experts attributed these perceived regional patterns to differences in humidity, housing characteristics and population mobility, and questionnaire respondents identified outdated acarological maps as a barrier to diagnosis; in this context, current international guidelines may not fully account for regional heterogeneity in mite exposure and sensitization (4, 6). Given the small qualitative sample and the unequal regional representation in the questionnaire, these regional patterns should be regarded as perceived and descriptive rather than as demonstrated differences. The environmental changes currently reshaping allergen distribution further supports the need to interpret mite allergy within a dynamic, environmentally informed framework.

Among specialists who systematically assessed sensitization to *B. tropicalis* or *L. destructor*, the reported rate of subsequent AIT prescription was high among this subgroup, suggesting close alignment between reported diagnostic recognition and therapeutic decision-making. This pattern is compatible with the hypothesis that a potential bottleneck may lie in diagnostic recognition and in the availability of environmental and regional exposure data, rather than in therapeutic decision-making once clinically relevant sensitization was reported. However, because eligibility required previous experience prescribing AIT, the high reported AIT use probably reflects, at least in part, the recruitment of AIT-experienced allergists and should not be generalized to all specialists. The reported preference for subcutaneous administration and the frequent use of polymerized extract mixtures describe the practice patterns observed among participating specialists only.

Previous *in vitro* studies have described the stability, allergenic potency and safety profile of polymerized mixtures (18, 19), and a limited number of clinical studies have reported reductions in symptoms and reliever medication use (20, 21). These data derive from previously published sources; the present survey describes reported prescribing practices only and should not be interpreted as validating the efficacy or superiority of any specific formulation. Expert consensus work, such as the Spanish REAL project, has considered well-justified mixtures of polymerized extracts an option for polysensitized patients, provided each component has clear clinical relevance and is present at a therapeutic concentration (14). Allergen immunotherapy products containing mite extracts are available both as individual extracts and as mixtures with other allergenic extracts, and allergen extracts are manufactured and standardized in accordance with applicable European requirements (22).

Interpretation of co-sensitization should account for cross-reactivity among *Dermatophagoides* spp., *B. tropicalis* and *L. destructor*, while recognizing that species-specific sensitization may also occur and should be considered when establishing clinical relevance and selecting AIT composition (4, 23). Current guidelines continue to endorse single-allergen extracts as the best-evidenced option for monosensitized patients (11), whereas polymerised mixtures should be reserved for genuinely polysensitized patients in whom each component has demonstrated clinical relevance (14). The main barriers to optimal AIT implementation identified by respondents suggest that even where therapeutic practice is well established, its further consolidation depends on the availability of stronger scientific evidence and clearer regulatory frameworks.

The results of this study may have several implications for clinical practice, which should be regarded as hypothesis-generating. The reported pattern, in which differences were observed mainly in diagnostic testing rather than in reported therapeutic decisions, suggests that diagnostic strategies for *B. tropicalis* and *L. destructor* merit further evaluation in geographical areas where these mites may be clinically relevant. However, the present data do not demonstrate that broader testing would improve diagnosis or treatment outcomes; this hypothesis requires confirmation in prospective, patient-level studies with objective assessment of clinical relevance. The findings are also consistent with a more individualized and context-driven diagnostic approach. While geographic factors remain an important starting point, clinicians may consider patient-specific exposure history, clinical trajectory, and the global environmental context, moving toward a more personalized, environmentally informed model of care. Furthermore, the frequent reporting of clinically relevant co-sensitization to *Dermatophagoides spp*. with other mites suggests that the scope of testing panels warrants consideration, particularly where allergen extract mixtures are used, as proposed by previous expert consensus (14). Given that sensitization to non-pyroglyphid mites such as *L. destructor* can reach high prevalences in some European regions and that *B. tropicalis* is increasingly recognized in subtropical and temperate climates, their exclusion from routine testing panels may contribute to potential gaps in diagnostic assessment in specific geographic areas and could, as a hypothesis to be confirmed, result in suboptimal treatment (4, 8, 16, 17, 23). These considerations suggest that future updates of clinical guidelines and diagnostic algorithms could take into account the evolving epidemiological landscape of mite sensitization, incorporating updated environmental, acarological, and regional exposure data into clinical decision-making tools.

This study has several strengths. It is based on a relatively large sample of clinicians across Spain and Portugal with extensive clinical experience in the field of mite allergy, providing a broad exploratory overview of real-world clinical practice. The integration of quantitative questionnaire data with qualitative insights from an Extended SC adds depth to the analysis and allows for a more contextualized interpretation of the findings. However, this study has several limitations that should be considered when interpreting the findings. Participants were recruited through a voluntary, non-probability convenience sample from a database maintained by Adelphi Targis on behalf of LETI Pharma, and eligibility required previous experience prescribing AIT. Together with the industry-sponsored context, this may have preferentially included clinicians more familiar with or interested in mite AIT and polymerized extract mixtures, potentially introducing selection or interest bias. Accordingly, the present findings describe the reported practice of AIT-experienced allergists accessible through the sponsor-associated contact database, rather than routine practice among all allergists in Spain and Portugal. In particular, the high proportions of reported AIT prescription and polymerized-mixture use may partly reflect this recruitment strategy and should not be generalized to all specialists. All outcomes were clinician-reported and were not validated against patient-level records, prescription data or diagnostic procedures. Also, the clinical relevance of sensitization was reported by the participating allergists and was not independently confirmed by standardized criteria, provocation testing or clinical outcome data. The questionnaire was not designed to compare the effectiveness, safety or superiority of specific formulations. The sample also included a limited number of Portuguese specialists and heterogeneous participation across regions, which constrains the representativeness of Portugal and of potentially underrepresented territories. Likewise, the regional patterns derived from the interviews rely on the perceptions of eight experts and should be regarded as perceived rather than demonstrated differences. Accordingly, the findings should be interpreted as hypothesis-generating descriptions of reported practice among participating specialists, rather than as evidence of comparative effectiveness, superiority or endorsement of any specific formulation. Nonetheless, the consistency between questionnaire findings and interview themes, and the convergence with external evidence on the presence of *B. tropicalis* and *L. destructor* in domestic dust in Spain and Portugal, support the internal consistency of the main descriptive findings.

Future research should focus on refining region-specific diagnostic strategies for *L. destructor* and *B. tropicalis*. In complex or polysensitized patients, component-resolved diagnostics and, when clinically indicated, allergen provocation testing may help establish the clinical relevance of sensitization and guide AIT composition. Diagnostic algorithms should integrate clinical history, regional exposure and molecular or extract-based testing rather than rely on sensitization profiles alone. Additional priorities include reinforcing environmental-control education and developing large-scale patient databases to support more robust regional evidence.

Integrating clinical, environmental and sensitization data, potentially supported by machine-learning approaches, could strengthen real-world evidence and inform more personalized AIT strategies. Future work should include epidemiological studies and clinical trials in patients sensitized to multiple mite species, together with updated region-specific acarological and environmental exposure maps to better tailor diagnostic panels and AIT formulations to local exposure patterns in Spain and Portugal (4, 7, 17).

## Conclusions

Among participating allergists in Spain and Portugal, routine testing was reported more frequently for *L. destructor* than for *B. tropicalis*, and respondents perceived the recognition of these mites in specific areas as a potential unmet need. Among participating specialists, once clinically relevant sensitization was reported, AIT prescription was frequently reported, predominantly by the subcutaneous route, among the subgroups of respondents who routinely tested the respective mite; this finding may partly reflect the recruitment of AIT-experienced allergists. Taken together, these findings are compatible with the hypothesis that the main limitation may lie upstream, in the detection of relevant sensitization. However, as the study is descriptive and based on self-reported practice, it does not demonstrate that broader testing for these mites would improve diagnosis or treatment, which should be addressed in prospective studies with objective confirmation of clinical relevance. These conclusions primarily reflect the participating specialists and clinical settings represented. The convenience-based recruitment strategy and potential selection and interest bias should be considered when interpreting their generalizability to allergists across Spain and Portugal.

## Data Availability

The raw data supporting the conclusions of this article will be made available by the authors, without undue reservation.
