# Supplementary material for: Shifting patterns of mite sensitization in Spain and Portugal: real-world diagnostic and immunotherapy practices (MITE-WISE project)
Source: Front Allergy. 2026 Oct 28;7:1946434. doi: 10.3389/falgy.2026.1946434 (PMC13751442; doi:10.3389/falgy.2026.1946434)
Supplement: Supplementary file 1 [file Table1.docx]

Supplementary Material

**Supplementary 1:**

**Questionnaire**

**Introduction**

Thank you very much for your interest in taking part in the **MITE-WISE** Project **(*Mite Immunotherapy Expert Wisdom & Evidence*)**.

As we have already mentioned, the aim of this project is **to understand the current management of sensitisation and allergy to *Blomia tropicalis (B. tropicalis)* and/or *Lepidoglyphus destructor* (*L. destructor)* in patients who are also allergic to *Dermatophagoides spp.* in Spain and Portugal,** taking into account the distinctive characteristics of these regions and their impact on the prevalence of these mites.

The ultimate aim of the project is to produce a document setting out **recommendations to improve the diagnosis and treatment of allergy to *B. tropicalis* and/or *L. destructor* in patients who are also allergic to *Dermatophagoides spp.***, based on the experiences of a representative group of Spanish and Portuguese allergists.

To this end, a questionnaire has been drawn up and validated by a scientific committee comprising four experts in allergology.

The questionnaire is structured into three sections:

**SECTION 1. Characteristics and description of the participants**

**SECTION 2. Detection and diagnosis of dust mite allergy**

**SECTION 3. Etiological treatment with allergen immunotherapy (AIT) in patients allergic to house dust mites**

There are no right or wrong answers, so please feel free to express your views and answer the questions based on your experience and professional judgement.

Please remember that you must complete the questionnaire by [insert date based on the survey launch date]

The estimated time to complete the questionnaire is 40 minutes.

**Once again, thank you for your time and involvement.**

**Terms and conditions of participation**

**Clause 1. Consent to participate**

The HEALTHCARE PROFESSIONAL has been informed about the **MITE-WISE Project (*Mite Immunotherapy Expert Wisdom & Evidence*)** (hereinafter, the “Project”), which is being carried out by Adelphi Targis, SL (ADELPHI TARGIS) on behalf of LETI Pharma, and has received and assessed the information relating to the Project.

The HEALTHCARE PROFESSIONAL agrees to take part in this project, the aim of which is **to understand the current practice regarding the management of sensitisation and allergy to *B. tropicalis* and/or *L. destructor* where there is also an allergy to *D. pteronyssinus and/or D. farinae*, in Spain and Portugal**

The HEALTHCARE PROFESSIONAL’s participation does not give rise to any conflict of interest, either directly or indirectly, nor does it breach any commitment entered into by the HEALTHCARE PROFESSIONAL or a close family member, particularly with third parties, and is not limited to their obligations towards the public administration. The HEALTHCARE PROFESSIONAL’s participation shall not have, nor has it had, any influence whatsoever on any business matter, purchasing or procurement decision, pricing, etc., particularly in relation to LETI Pharma’s products, nor shall it entail any obligation on the part of the HEALTHCARE PROFESSIONAL to refer any patient, doctor or healthcare organisation to LETI Pharma’s products or services.

**Clause 2. Confidentiality**

All information that the HEALTHCARE PROFESSIONAL acquires in any way as a result of their participation in the Project, including any information relating to LETI Pharma that links the HEALTHCARE PROFESSIONAL to ADELPHI TARGIS, shall be kept confidential and treated by the HEALTHCARE PROFESSIONAL, even after the completion of the Project, with such care as is reasonably necessary and practicable to prevent such information from being disclosed to third parties.

The HEALTHCARE PROFESSIONAL expressly undertakes to use such information solely and exclusively for the proper conduct of the Project. The obligations of secrecy and confidentiality shall remain in force and shall be enforceable throughout the term of the Project and for a period of ten (10) years following the date of completion of the Project.

**Clause 3. Protection of personal data**

The HEALTHCARE PROFESSIONAL acknowledges and accepts that their personal data included in this project and any data that may subsequently be obtained in connection with it are processed by ADELPHI TARGIS, in accordance with Regulation (EU) 2016/679 or the General Data Protection Regulation (GDPR), for the purpose of facilitating the maintenance, fulfilment, development, monitoring and management of this contractual relationship and any that may arise in the future. The legal basis for the use of the data shall be the Healthcare Professional’s consent, in addition to the existing contractual relationship between the parties.

Furthermore, the HEALTHCARE PROFESSIONAL is aware that other bodies (authorities, courts) may access their data when they need to verify it in accordance with current legislation. In addition, personal data may be processed by service providers (for example, IT services) where they have signed a Data Processing Agreement (DPA). Your data may be transferred to third parties located outside the European Economic Area (EEA), but in such cases, only to those countries with a similar level of data protection approved by the European Commission or , subject to a prior agreement that will include the Standard Contractual Clauses (SCCs) approved by the European Commission.

The HEALTHCARE PROFESSIONAL’s personal data will be retained for the minimum period necessary to fulfil the purposes for which it was collected, in accordance with current legislation, and for as long as the HEALTHCARE PROFESSIONAL does not request its erasure.

The HEALTHCARE PROFESSIONAL is aware that ADELPHI TARGIS guarantees that, to the extent possible under applicable regulations, they may exercise their rights of access, rectification, erasure, restriction, objection and data portability, as well as revoke the consent initially given, by sending a written request to Calle Aribau, 230–240, 8th Floor, Unit X, 08006 Barcelona, quoting the reference ‘Personal Data Protection’, or by email to: dpo@adelphitargis.com.

Furthermore, the HEALTHCARE PROFESSIONAL is aware of the possibility of exercising their rights or lodging a complaint with the relevant supervisory authority, namely the Spanish Data Protection Agency (AEPD).

**Clause 4. Industrial and intellectual property**

The HEALTHCARE PROFESSIONAL grants ADELPHI TARGIS and, in turn, LETI Pharma, including any other entities forming part of LETI Pharma, the exclusive rights to use and exploit, in any form and by any means, whether known or future, the deliverables provided by the HEALTHCARE PROFESSIONAL (hereinafter, the “Material”).

The scope of the assignment of the Material covers all countries worldwide and for the maximum term provided for under current legislation on intellectual property, without prejudice to any inherent moral rights that may attest to the HEALTHCARE PROFESSIONAL’s authorship.

With the exception of their moral rights, the HEALTHCARE PROFESSIONAL may not demand or claim any right of ownership or use in respect of intellectual and industrial property rights during the term of the Project or after its completion.

**[P0]** By taking part in this survey, the HEALTHCARE PROFESSIONAL agrees to participate in this project and declares that:

They acknowledge that they possess sufficient academic and professional qualifications to carry out the project by completing the survey.

They have received the project information and have been informed about the implementation of the MITE-WISE Project

P.1. **[P1]** I confirm that I have read the terms and conditions of the Project (participation consent clause, confidentiality clause, personal data protection clause, industrial and intellectual property clause) and agree to them.

- **[1] Please tick the box to give your consent.**

# 1 Form registration:

Please complete the following fields with the required details:

P.2. **[P2]** Please enter your first name and surname

**[P2_1] First name: ________________________________________________________**

**[P2_2] Surname 1: _______________________________________________________**

**[P2_3] Surname 2: _______________________________________________________**

P.3. **[P3]** Please provide your main contact email address

**Email address: _______________________________________________**

P.4. **[P4] In which country do you practise medicine?**

- **[1]** Spain
- **[2]** Portugal

P.5. **[P5]** What is your medical specialism?

- **[1]** Allergy specialist
- [**P5_OTROS**] Other

# 2. Questionnaire

[Comments regarding the questionnaire’s structure are shown in orange]

## 1.1 Characteristics and description of participants

*Participant’s sociodemographic data*

P.6. **[P6]** Please state your **place of work and town/city**:

If you work at more than one workplace, please provide a single answer based on the workplace where you carry out the majority of your professional activities.

**[P6_1]** Place of work: _____________

**[P6_2]** Town/city: __________

P.7. **[P7]** The centre/hospital where you carry out your **professional work** is a …

Please tick one answer only, thinking of the type of centre where you carry out the majority of your professional work.

- [1] Peripheral specialist centre
- [2] Private practice.
- [3] Small district hospital, with an average of fewer than 150 beds, with virtually no high-tech equipment, few doctors and a low level of clinical complexity.
- [4] Basic general hospital, medium-sized with fewer than 200 beds, minimal technological resources, with some teaching role and a slightly higher level of complexity in the cases treated.
- [5] Medium-sized regional hospital with around 500 beds. More than 50 MIR doctors and an average of 269 doctors. Medium complexity (1.5 complex departments and a case mix of 1.01).
- [6] A large hospital, but more varied in terms of staffing, size and activity. High level of teaching activity (more than 160 MIR doctors) and high complexity (an average of 4 complex departments and a case mix greater than 1.20).
- [7] A hospital of great structural importance and high activity levels. Full range of services. More than 680 doctors and around 300 MIRs. Includes large hospital complexes.

P.8. **[P8]** Please indicate **the percentage of your clinical work carried out in each of these areas out of your total working time:**

- Please enter your answer.
- **[P8_1]** _____ % Public Centre/Hospital
- **[P8_2]** _____% Private centre/hospital

[The total of your answers must add up to 100%]

P.9. **[P9]** Please indicate in which **Autonomous Community** you carry out your professional activity:

Please select only one answer.

- **[1]** Andalusia
- **[2]** Aragon
- **[3]** Asturias
- **[4]** Cantabria
- **[5]** Castile-La Mancha
- **[6]** Castile and León
- **[7]** Catalonia
- **[8]** Ceuta
- **[9]**  Community of Madrid
- [10] Chartered Community of Navarre
- **[11]** Valencian Community
- **[12]** Extremadura
- **[13]** Galicia
- **[14]** Balearic Islands
- **[15]** Canary Islands
- **[16]** La Rioja
- **[17]** Melilla
- **[18]** Basque Country
- **[19]** Region of Murcia

P.10. **[P10]** Please indicate in which **province** you carry out your professional activity:

Please select only one answer.

- **[1]** Albacete
- **[2]** Alicante/Alacant
- **[3]** Almería
- [4] Araba/Álava
- **[5]** Asturias
- **[6]** Ávila
- **[7]** Badajoz
- **[8]** Balearic Islands
- **[9]** Barcelona
- **[10]** Bizkaia/Vizcaya
- **[11]** Burgos
- **[12]** Cáceres
- **[13]** Cádiz
- **[14]** Cantabria
- **[15]** Castellón/Castelló
- **[16]** Ciudad Real
- **[17]** Córdoba
- **[18]** A Coruña
- **[19]** Cuenca
- **[20]** Gipuzkoa
- **[21]** Girona
- **[22]** Granada
- **[23]** Guadalajara
- **[24]** Huelva
- **[25]** Huesca
- **[26]** Jaén
- **[27]** León
- **[28]** Lleida
- **[29]** Lugo
- **[30]** Madrid
- **[31]** Málaga
- **[32]** Murcia
- **[33]** Navarre
- **[34]** Ourense
- **[35]** Palencia
- **[36]** Las Palmas
- **[37]** Pontevedra
- **[38]** La Rioja
- **[39]** Salamanca
- **[40]** Santa Cruz de Tenerife
- **[41]** Segovia
- **[42]** Seville
- **[43]** Soria
- **[44]** Tarragona
- **[45]** Teruel
- **[46]** Toledo
- **[47]** Valencia
- **[48]** Valladolid
- **[49]** Zamora
- **[50]** Zaragoza
- **[51]** Ceuta
- **[52]** Melilla

*Professional experience in the field of allergology*

P.11. **[P11]** How many years have you been practising as a specialist in allergology?

Please select only one answer, excluding your MIR training period

- **[1]** Less than 5 years
- **[2]** 5 to 10 years
- **[3]** 11 to 15 years
- **[4]** 16 to 20 years
- **[5]** More than 20 years

P.12. **[P12]** How many **years’ experience** do you have in **aetiological treatment with ITA based on mite extracts**?

Please tick one answer only, excluding your MIR training period

- **[1]** Less than 5 years
- **[2]** 5 to 10 years
- **[3]** 11 to 15 years
- **[4]** 16 to 20 years
- **[5]** More than 20 years

P.13. **[P13]** Have you been involved in any **studies or publications** on dust mite allergy?

Please tick only one answer.

- **[1]** Yes🡪 Show P14
- **[2]** No

P.14. **[P14] When was the most recent publication?**

Please enter your answer.

- Year of publication: _____________

(only numerical answers will be accepted)

## 1.2 Patient numbers and current practice

P.15. **[P15]** Considering your total current professional activity, approximately **what proportion of the patients you see on average each month are paediatric patients (< 14 years)**?

Please enter your answer (think in terms of patients rather than consultations, and base your answer on a percentage for a month that is representative of the rest of the year)

- **[P15_1]** _____ % Patients under 14 years of age
- **[P15_2]** _____% Patients aged ≥14 years

[The total of the answers must add up to 100%]

P.16. **[P16]** Considering your current professional practice as a whole, approximately **what percentage of the patients you see on average each month are patients with a dust mite allergy**?

Please enter your answer (think in terms of patients rather than consultations, and base your answer on a percentage for a month that is representative of the rest of the year).

- _____ % Patients with dust mite allergies

P.17. **[P17]** Considering your current professional practice as a whole, approximately **what percentage of these patients with house dust mite allergies come from urban areas and what percentage come from rural areas, where ‘rural’ refers to areas associated with farming and/or livestock rearing**?

Please enter your answer (think in terms of patients rather than consultations, and base your answer on a percentage of one month that is representative of the rest of the year).

- **[P17_1]** _____ % of patients with house dust mite allergy who come from an urban environment
- **[P17_2]** _____ % Patients with house dust mite allergy from a rural environment
- **[P17_3]** _____ % Unknown

[The total of the answers must add up to 100%]

P.18. **[P18]** Considering your current professional practice as a whole, approximately **what percentage of these patients with house dust mite allergy exhibit symptoms associated with their occupational activities**?

Please enter your answer (think in terms of patients rather than consultations, and base your answer on a percentage of one month that is representative of the rest of the year).

- **[P18_1]** _____ % Patients with house dust mite allergy exhibiting symptoms associated with their professional activity
- **[P18_2]** _____ % Unknown or not related to their professional activity

[The total of the answers must add up to 100%]

P.19. **[P19]** Considering your current professional practice as a whole, approximately **what percentage of the patients you see on average each month have been diagnosed with an allergy to *Dermatophagoides spp.***?

Please enter your answer (think in terms of patients rather than consultations, and base your answer on a percentage for a month that is representative of the rest of the year).

- _____ % Patients with an allergy to ***Dermatophagoides spp.***

P.20. **[P20]** Of all these patients with an allergy to ***Dermatophagoides spp.***, **what percentage of patients present with the following allergic symptoms**?

Please give your answer, *bearing in mind that several of these symptoms may sometimes coexist in the same patient* (think of patients outside of consultations and answer based on the percentage for a month that is representative of the rest of the year).

- **[P20_1]** _____ % Patients with allergic asthma (AA)
- **[P20_2]** _____% Patients with allergic rhinitis/rhinoconjunctivitis (AR/ARC)
- **[P20_3]** _____ % Patients with atopic dermatitis (AD)

P.21. **[P21]** Of all your patients with AA caused by ***Dermatophagoides spp.*,** **what is the severity of their symptoms, according to the scale set out in the GEMA guidelines**?

Please enter your answer (think in terms of patients rather than consultations, and base your answer on a percentage of one month that is representative of the rest of the year).

- **[P21_1]** _____ % Patients with intermittent AA
- **[P21_2]** _____ % Patients with mild persistent AA
- **[P21_3]** _____ % Patients with moderate persistent AA
- **[P21_4]** _____ % Patients with severe persistent AA

[The total of the responses must add up to 100%]

P.22. **[P22]** Of all your patients with RA/RCA caused by ***Dermatophagoides spp.*, what is the severity of their symptoms according to the scale set out in the ARIA guidelines**?

Please enter your answer (think in terms of patients rather than consultations, and base your answer on a percentage of one month that is representative of the rest of the year).

- **[P22_1]** _____ % Patients with mild intermittent AR/RCA
- **[P22_2]** _____ % of patients with moderate-to-severe intermittent RA/RCA
- **[P22_3]** _____ % Patients with mild persistent RA/RCA
- **[P22_4]** _____ % Patients with moderate-to-severe persistent RA/RCA

[The total of the responses must add up to 100%]

P.23. **[P23]** Of all your patients with DA caused by ***Dermatophagoides spp.*, what is the severity of their symptoms according to the EASI scale**?

Please enter your answer (think in terms of patients rather than consultations, and base your answer on a percentage of one month that is representative of the rest of the year).

- **[P23_1]** _____ % Patients with mild AD: EASI <7.0
- **[P23_2]** _____ % Patients with moderate AD: EASI 7.1–21
- **[P23_3]** _____ % Patients with severe DA: EASI 21.1–50
- **[P23_4]** _____ % Patients with very severe DA: EASI 50.1–72

[The total of the responses must add up to 100%]

P.24. **[P24]** Of all your patients with an allergy to ***Dermatophagoides spp.***, **what is the percentage of patients receiving aetiological treatment with ITA for *Dermatophagoides spp.*?**

Please enter your answer (think in terms of patients rather than consultations, and base your answer on a percentage for a month that is representative of the rest of the year).

- _____ % of patients receiving aetiological treatment with ITA for *Dermatophagoides spp.*

P.25. **[P25]** Considering your current professional practice as a whole, approximately **what percentage of the patients you see on average each month have been diagnosed with an allergy to *Dermatophagoides spp.* and *B. tropicalis***?

Please enter your answer (think in terms of patients rather than consultations, and base your answer on a percentage for a month that is representative of the rest of the year).

- _____ % Patients with an allergy to *Dermatophagoides spp.* and *B. tropicalis*

P.26. **[Q26]** Of the total number of these patients with an allergy to *Dermatophagoides spp.* and *B. tropicalis*, **what percentage of patients present with the following allergic symptoms**?

Please enter your answer (think in terms of patients rather than consultations, and base your answer on a percentage for a month that is representative of the rest of the year).

- **[P26_1]** _____ % Patients with AA
- **[P26_2]** _____ % Patients with AR/ACR
- **[P26_3]** _____ % Patients with DA

P.27. **[P27]** Of all your patients with AA caused by *Dermatophagoides spp.* and *B. tropicalis*, **what is the severity of their symptoms according to the scale set out in the GEMA guidelines**?

Please enter your answer (think in terms of patients rather than consultations, and base your answer on a percentage of one month that is representative of the rest of the year).

- **[P27_1]** _____ % Patients with intermittent AA
- **[P27_2]** _____ % Patients with mild persistent AA
- **[P27_3]** _____ % Patients with moderate persistent AA
- **[P27_4]** _____ % Patients with severe persistent AA

[The total of the responses must add up to 100%]

P.28. **[P28]** Of all your patients with RA/RCA caused by *Dermatophagoides spp.* and *B. tropicalis***, what is the severity of their symptoms according to the scale set out in the ARIA guidelines**?

Please enter your answer (think in terms of patients rather than consultations, and base your answer on a percentage of one month that is representative of the rest of the year).

- **[P28_1]** _____ % Patients with mild intermittent AR/AC
- **[P28_2]** _____ % Patients with moderate-to-severe intermittent AR/RCA
- **[P28_3]** _____ % Patients with mild persistent RA/RCA
- **[P28_4]** _____ % Patients with moderate-to-severe persistent RA/RCA

[The total of the responses must add up to 100%]

P.29. **[P29]** Of all your patients with DA caused by *Dermatophagoides spp.* and *B. tropicalis***, what is the severity of their symptoms according to the EASI scale**?

Please enter your answer (think in terms of patients rather than consultations, and base your answer on a percentage of one month that is representative of the rest of the year).

- **[P29_1]** _____ % Patients with mild AD: EASI <7.0
- **[P29_2]** _____ % Patients with moderate AD: EASI 7.1–21
- **[P29_3]** _____ % Patients with severe DA: EASI 21.1–50
- **[P29_4]** _____ % Patients with very severe AD: EASI 50.1–72

[The total of the responses must add up to 100%]

P.30. **[P30]** Considering your current professional practice as a whole, approximately **what percentage of the patients you see on average each month have been diagnosed with an allergy to *Dermatophagoides spp.* and *L. destructor***?

Please enter your answer (think in terms of patients rather than consultations, and base your answer on a percentage of one month that is representative of the rest of the year).

- _____ % of patients with an allergy to *Dermatophagoides spp.* and *L. destructor*

P.31. **[P31]** Of all your patients with an allergy to *Dermatophagoides spp.* and *L. destructor*, **what percentage of these patients present with the following allergic symptoms**?

Please enter your answer (think in terms of patients rather than consultations, and base your answer on a percentage for a month that is representative of the rest of the year).

- **[P31_1]** _____ % Patients with AA
- **[P31_2]** _____ % Patients with RA/RCA
- **[P31_3]** _____ % Patients with AD

P.32. **[P32]** Of all your patients with AA caused by *Dermatophagoides spp.* and *L. destructor*, **what is the severity of their symptoms according to the scale set out in the GEMA guidelines**?

Please enter your answer (think in terms of patients rather than consultations, and base your answer on a percentage of one month that is representative of the rest of the year).

- **[P32_1]** _____ % Patients with intermittent AA
- **[P32_2]** _____ % Patients with mild persistent AA
- **[P32_3]** _____ % Patients with moderate persistent AA
- **[P32_4]** _____ % Patients with severe persistent AA

[The total of the responses must add up to 100%]

P.33. **[P33]** Of all your patients with AR/RCA caused by *Dermatophagoides spp.* and *L. destructor***, what is the severity of their symptoms according to the scale set out in the ARIA guidelines**?

Please enter your answer (think in terms of patients rather than consultations, and base your answer on a percentage of one month that is representative of the rest of the year).

- **[P33_1]** _____ % Patients with mild intermittent RA/RCA
- **[P33_2]** _____ % Patients with moderate-to-severe intermittent RA/RCA
- **[P33_3]** _____ % Patients with mild persistent RA/RCA
- **[P33_4]** _____ % Patients with moderate-to-severe persistent RA/RCA

[The total of the responses must add up to 100%]

P.34. **[P34]** Of all your patients with DA caused by *Dermatophagoides spp.* and *L. destructor***, what is the severity of their symptoms according to the EASI scale**?

Please enter your answer (think in terms of patients rather than consultations, and base your answer on a percentage of one month that is representative of the rest of the year).

- **[P34_1]** _____ % Patients with mild AD: EASI <7.0
- **[P34_2]** _____ % Patients with moderate AD: EASI 7.1–21
- **[P34_3]** _____ % Patients with severe DA: EASI 21.1–50
- **[P34_4]** _____ % Patients with very severe DA: EASI 50.1–72

[The total of the responses must add up to 100%]

## 1.3 Diagnosis of dust mite allergy

*Frequency and determinants in the diagnosis of allergy*

P.35. **[P35]** In patients with an allergy to ***Dermatophagoides spp.,*** do you **always** carry out a **sensitisation test for *B. tropicalis***?

Please tick only one answer.

- **[1]** Yes
- **[2]** No

P.36. **[P36]** Please rank the following attributes in order of **clinical relevance when carrying out a *B. tropicalis* sensitisation test** in patients with an allergy to *Dermatophagoides spp.*

Rank them from 1 to 10, with 1 being the most relevant and 10 the least relevant

- **[P36_1]** Geographical area of origin
- **[P36_2]** Geographical area of residence
- **[P36_3]** Frequency of travel to hot and humid areas
- **[P36_4]** Occupational activity and/or characteristics
- **[P36_5]** Household characteristics
- **[P36_6]** Leisure activities in hot and humid environments
- **[P36_7]** Clinical pattern of symptoms (time of onset, frequency, association with specific daily routines, cyclical nature, etc.)
- **[P36_8]** Lack of symptom control (despite standard treatment and specific recommendations)
- **[P36_9]** Failure of/response to previous treatment with ITA
- **[P36_10]** Other?:
  - **P36_OTHERS____________**

P.37. **[P37]** Please rate these same attributes on a scale of 1 to 10 according to their **clinical relevance when conducting a *B. tropicalis* sensitisation test** in patients with an allergy to *Dermatophagoides spp.*

Rate from 1 to 10, where 1 is not at all important and 10 is very important

|  | **Not at all important** |  |  |  |  |  |  |  |  | **Very important** |
| --- | --- | --- | --- | --- | --- | --- | --- | --- | --- | --- |
|  | **1** | **2** | **3** | **4** | **5** | **6** | **7** | **8** | **9** | **10** |
| **[P37_1]** Geographical area of origin | □ | □ | □ | □ | □ | □ | □ | □ | □ | □ |
| **[P37_2]** Geographical area of residence | □ | □ | □ | □ | □ | □ | □ | □ | □ | □ |
| **[P37_3]**. Frequency of trips to hot and humid areas | □ | □ | □ | □ | □ | □ | □ | □ | □ | □ |
| **[P37_4]** Occupational activity and/or characteristics | □ | □ | □ | □ | □ | □ | □ | □ | □ | □ |
| **[P37_5]** Household characteristics | □ | □ | □ | □ | □ | □ | □ | □ | □ | □ |
| **P37_6]** Recreational activities in warm and humid environments | □ | □ | □ | □ | □ | □ | □ | □ | □ | □ |
| **[P37_7]** Clinical pattern of symptoms (time of onset, frequency, association with specific daily routines, cyclical nature, etc.) | □ | □ | □ | □ | □ | □ | □ | □ | □ | □ |
| **[P37_8]** Inadequate symptom control (despite standard treatment and specific recommendations) | □ | □ | □ | □ | □ | □ | □ | □ | □ | □ |
| **[P37_9]** Failure/response to previous treatment with ITA | □ | □ | □ | □ | □ | □ | □ | □ | □ | □ |
| **[P37_10]** Other | □ | □ | □ | □ | □ | □ | □ | □ | □ | □ |

P.38. **[Q38]** What **characteristics of the geographical area of exposure** do you think need to be taken into account when evaluating **the study with *B. tropicalis*** in patients with an allergy to *Dermatophagoides spp.*?

Please tick ALL the answers that apply

- **[P38_1]** Humidity
- **[P38_2]** Temperature
- **[P38_3]** Rural environment (related to agricultural and/or livestock farming activities)
- [**P38_OTHERS**] Other
  - **____________**

P.39. **[P39]** How often do you carry out each of the following **diagnostic tests** to assess **sensitisation to *B. tropicalis*** in patients with an allergy to *Dermatophagoides spp.*?

Rate on a scale of 0 to 9, where 0 means ‘never’ and 9 means ‘always’

|  | **Never** |  |  |  |  |  |  |  |  | **Always** |
| --- | --- | --- | --- | --- | --- | --- | --- | --- | --- | --- |
|  | **0** | **1** | **2** | **3** | **4** | **5** | **6** | **7** | **8** | **9** |
| **[P39_1]** Medical history, | □ | □ | □ | □ | □ | □ | □ | □ | □ | □ |
| **[P39_2]** Physical examination | □ | □ | □ | □ | □ | □ | □ | □ | □ | □ |
| **[P39_3]**. SPT (Prick test) | □ | □ | □ | □ | □ | □ | □ | □ | □ | □ |
| **[P39_4]** Provocation tests | □ | □ | □ | □ | □ | □ | □ | □ | □ | □ |
| **P39_5]** *In vitro* tests: full extract | □ | □ | □ | □ | □ | □ | □ | □ | □ | □ |
| **[P39_6]** *In vitro* tests*:*  component-based diagnosis | □ | □ | □ | □ | □ | □ | □ | □ | □ | □ |
| **[P39_7]** Identification of mites present in the patient’s home | □ | □ | □ | □ | □ | □ | □ | □ | □ | □ |
| **[P39_8]** Other | □ | □ | □ | □ | □ | □ | □ | □ | □ | □ |

P.40. **[P40]** In patients with an allergy to ***Dermatophagoides spp.***, do you **always** carry out a **sensitivity test for *L. destructor***?

Please select only one answer.

- **[1]** Yes
- **[2]** No

P.41. **[P41]** Please rank the following attributes in order of **clinical relevance when conducting a *L. destructor* sensitisation test** in patients with an allergy to *Dermatophagoides spp.*

- Rank them from 1 to 10, with 1 being the most relevant and 10 the least relevant
- **[P41_1]** Geographical area of origin
- **[P41_2]** Geographical area of residence
- **[P41_3]** Frequency of travel to hot and humid areas
- **[P41_4]** Occupational activity and/or characteristics
- **[P41_5]** Household characteristics
- **[P41_6]** Leisure activities in hot and humid environments
- **[P41_7]** Clinical pattern of symptoms (time of onset, frequency, association with specific daily routines, cyclical nature, etc.)
- **[P41_8]** Lack of symptom control (despite standard treatment and specific recommendations)
- **[P41_9]** Failure of/response to previous treatment with ITA
- **[P41_10]** Any others?

**[P41_OTHERS]____________**

P.42. **[P42]** Please rate these same attributes on a scale of 1 to 10 according to their **clinical relevance when conducting a *L. destructor* sensitisation study** in patients with an allergy to *Dermatophagoides spp.*

Rate from 1 to 10, where 1 is not at all important and 10 is very important

|  | **Not at all important** |  |  |  |  |  |  |  |  | **Very important** |
| --- | --- | --- | --- | --- | --- | --- | --- | --- | --- | --- |
|  | **1** | **2** | **3** | **4** | **5** | **6** | **7** | **8** | **9** | **10** |
| **[P42_1]** Geographical area of origin | □ | □ | □ | □ | □ | □ | □ | □ | □ | □ |
| **[P42_2]** Geographical area of residence | □ | □ | □ | □ | □ | □ | □ | □ | □ | □ |
| **[P42_3]**. Frequency of trips to hot and humid areas | □ | □ | □ | □ | □ | □ | □ | □ | □ | □ |
| **[P42_4]** Occupational activity and/or characteristics | □ | □ | □ | □ | □ | □ | □ | □ | □ | □ |
| **[P42_5]** Household characteristics | □ | □ | □ | □ | □ | □ | □ | □ | □ | □ |
| **[P42_6]** Recreational activities in warm and humid environments | □ | □ | □ | □ | □ | □ | □ | □ | □ | □ |
| **[P42_7]** Clinical pattern of symptoms (time of onset, frequency, association with specific daily routines, cyclical nature, etc.) | □ | □ | □ | □ | □ | □ | □ | □ | □ | □ |
| **[P42_8]** Inadequate symptom control (despite standard treatment and specific recommendations) | □ | □ | □ | □ | □ | □ | □ | □ | □ | □ |
| **[P42_9]** Failure/response to previous treatment with ITA | □ | □ | □ | □ | □ | □ | □ | □ | □ | □ |
| **[P42_10]** Other | □ | □ | □ | □ | □ | □ | □ | □ | □ | □ |

P.43. **[P43**] What characteristics of the **geographical area of exposure** do you think need to be taken into account when evaluating **the study with *L. destructor*** in patients with an allergy to *Dermatophagoides spp.*?

Please tick ALL the answers that apply

- **[P43_1]** Humidity
- **[P43_2]** Temperature
- **[P43_3]** Rural environment (related to agricultural and/or livestock farming activities)
- **[P43_OTHERS]** Other
  - **______________**

P.44. **[P44]** How often do you carry out each of the following **diagnostic tests** to assess **sensitisation to *L. destructor*** in patients with an allergy to *Dermatophagoides spp.*?

Rate on a scale of 0 to 9, where 0 means ‘never’ and 9 means ‘always’

|  | **Never** |  |  |  |  |  |  |  |  | **Always** |
| --- | --- | --- | --- | --- | --- | --- | --- | --- | --- | --- |
|  | **0** | **1** | **2** | **3** | **4** | **5** | **6** | **7** | **8** | **9** |
| **[P44_1]** Medical history, | □ | □ | □ | □ | □ | □ | □ | □ | □ | □ |
| **[P44_2]** Physical examination | □ | □ | □ | □ | □ | □ | □ | □ | □ | □ |
| **[P44_3]**. SPT (Prick test) | □ | □ | □ | □ | □ | □ | □ | □ | □ | □ |
| **[P44_4]** Provocation tests | □ | □ | □ | □ | □ | □ | □ | □ | □ | □ |
| **[P44_5]** *In vitro* tests: full extract | □ | □ | □ | □ | □ | □ | □ | □ | □ | □ |
| **[P44_6]** *In vitro* tests*:* component-based diagnosis | □ | □ | □ | □ | □ | □ | □ | □ | □ | □ |
| **[P44_7]** Identification of mites present in the patient’s home | □ | □ | □ | □ | □ | □ | □ | □ | □ | □ |
| **[P44_8]** Other | □ | □ | □ | □ | □ | □ | □ | □ | □ | □ |

*Barriers and opportunities for detection and diagnosis*

P.45. **[P45]** Please rank the following factors according to **the extent to which they hinder the detection and diagnosis of allergy to *B. tropicalis*** in patients with allergy to ***Dermatophagoides spp.***

Rank them from 1 to 11, with 1 being the most relevant and 11 the least relevant

- **[P45_1]** Lack of data on sensitisation profiles
- **[P45_2]** Availability of provocation tests
- **[P45_3]** Availability of *in vivo* tests
- **[P45_4]** Availability of *in vitro* tests
- **[P45_5]** Sensitivity of the tests
- **[P45_6]** Clinical relevance in your region
- **[P45_7]** Lack of mention in existing guidelines
- **[P45_8]** Lack of guidelines/protocols suited to the reality of the patient and their environment
- **[P45_9]** Difficulty in obtaining an up-to-date acarological survey
- **[P45_10]** Lack of an acarological analysis and characterisation of the patient’s home
- **[P45_10]** Belief that ITA against *Dermatophagoides spp.* is sufficient
- **[P45_12]** Other
  - **P45_OTHERS ______________**

P.46. **[P46]** Please rate these same factors on a scale of 1 to 10 according to **the extent to which they hinder the detection and diagnosis of allergy to *B. tropicalis*** in patients with allergy to *Dermatophagoides spp.*

Rate on a scale of 1 to 10, where 1 is not at all significant and 10 is very significant

|  | **Not at all important** |  |  |  |  |  |  |  |  | **Very significant** |
| --- | --- | --- | --- | --- | --- | --- | --- | --- | --- | --- |
|  | **1** | **2** | **3** | **4** | **5** | **6** | **7** | **8** | **9** | **10** |
| **[P46_1]** Lack of sensitisation profile data | □ | □ | □ | □ | □ | □ | □ | □ | □ | □ |
| **[P46_2]** Availability of provocation tests | □ | □ | □ | □ | □ | □ | □ | □ | □ | □ |
| **[P46_3]** Availability of in vivo tests | □ | □ | □ | □ | □ | □ | □ | □ | □ | □ |
| **[P46_4]** Availability of in vitro tests | □ | □ | □ | □ | □ | □ | □ | □ | □ | □ |
| **[P46_5]** Test sensitivity | □ | □ | □ | □ | □ | □ | □ | □ | □ | □ |
| **[P46_6]** Clinical relevance in your area | □ | □ | □ | □ | □ | □ | □ | □ | □ | □ |
| **[P46_7]** Not mentioned in existing guidelines | □ | □ | □ | □ | □ | □ | □ | □ | □ | □ |
| **[P46_8]** Lack of guidelines/protocols suited to the patient’s actual situation and their environment | □ | □ | □ | □ | □ | □ | □ | □ | □ | □ |
| **[P46_9]** Difficulty in obtaining an up-to-date acarological map | □ | □ | □ | □ | □ | □ | □ | □ | □ | □ |
| **[P46_10] Failure to carry out an acarological analysis and characterisation of the patient’s home** | □ | □ | □ | □ | □ | □ | □ | □ | □ | □ |
| **[P46_11]** The misconception that ITA against *Dermatophagoides spp.* is sufficient | □ | □ | □ | □ | □ | □ | □ | □ | □ | □ |
| **[P46_12]** Other | □ | □ | □ | □ | □ | □ | □ | □ | □ | □ |

P.47. **[P47]** Please rank the following factors according to **the extent to which they hinder the detection and diagnosis of *L. destructor* allergy** in patients with *Dermatophagoides spp.* allergy*.*

- Rank them from 1 to 11, with 1 being the most relevant and 11 the least relevant
- **[P47_1]** Lack of data on sensitisation profiles
- **[P47_2]** Availability of provocation tests
- **[P47_3]** Availability of *in vivo* tests
- **[P47_4]** Availability of *in vitro* tests
- **[P47_5]** Sensitivity of the tests
- **[P47_6]** Clinical relevance in your region
- **[P47_7]** Lack of mention in existing guidelines
- **[P47_8]** Lack of guidelines/protocols suited to the reality of the patient and their environment
- **[P47_9]** Difficulty in obtaining an up-to-date acarological survey
- **[P47_10]** Lack of an acarological analysis and characterisation of the patient’s home
- **[P47_11]** Belief that ITA against *Dermatophagoides spp.* is sufficient
- **[P47_12]** Other
  - **P47_OTHERS ______________**

P.48. **[P48]** Please rate these same factors on a scale of 1 to 10 according to **the extent to which they hinder the detection and diagnosis of *L. destructor* allergy** in patients with an allergy to *Dermatophagoides spp.*

Rate on a scale of 1 to 10, where 1 is not at all significant and 10 is very significant

|  | **Not at all important** |  |  |  |  |  |  |  |  | **Very significant** |
| --- | --- | --- | --- | --- | --- | --- | --- | --- | --- | --- |
|  | **1** | **2** | **3** | **4** | **5** | **6** | **7** | **8** | **9** | **10** |
| **[P48_1]** Lack of sensitisation profile data | □ | □ | □ | □ | □ | □ | □ | □ | □ | □ |
| **[P48_2]** Availability of provocation tests | □ | □ | □ | □ | □ | □ | □ | □ | □ | □ |
| **[P48_3]** Availability of in vivo tests | □ | □ | □ | □ | □ | □ | □ | □ | □ | □ |
| **[P48_4]** Availability of in vitro tests | □ | □ | □ | □ | □ | □ | □ | □ | □ | □ |
| **[P48_5]** Test sensitivity | □ | □ | □ | □ | □ | □ | □ | □ | □ | □ |
| **[P48_6]** Clinical relevance in your area | □ | □ | □ | □ | □ | □ | □ | □ | □ | □ |
| **[P48_7]** Not mentioned in existing guidelines | □ | □ | □ | □ | □ | □ | □ | □ | □ | □ |
| **[P48_8]** Lack of guidelines/protocols suited to the patient’s circumstances and environment | □ | □ | □ | □ | □ | □ | □ | □ | □ | □ |
| **[P48_9]** Difficulty in obtaining an up-to-date acarological map | □ | □ | □ | □ | □ | □ | □ | □ | □ | □ |
| **[P48_10]** Failure to carry out an acarological analysis and characterisation of the patient’s home |  |  |  |  |  |  |  |  |  |  |
| **[P48_11]** The mistaken belief that ITA against *Dermatophagoides spp.* is sufficient | □ | □ | □ | □ | □ | □ | □ | □ | □ | □ |
| **[P48_12]** Other | □ | □ | □ | □ | □ | □ | □ | □ | □ | □ |

## 1.4 Treatment of dust mite allergy

*Frequency and determinants of allergy treatment*

P.49. **[P49]** Considering your current professional practice as a whole, approximately what percentage of the patients you see on average each month who have **been diagnosed with an allergy to *Dermatophagoides spp.* and *B. tropicalis* do you recommend etiological treatment with ITA against both mites**?

Please enter your answer (think in terms of patients rather than consultations, and base your answer on a percentage for a month that is representative of the rest of the year). If you do not see any patients with an allergy to Dermatophagoides spp. and B. tropicalis, please select the ‘Not applicable’ option.

________% of patients with allergies to *Dermatophagoides spp*. and *B. tropicalis* for whom you recommend aphidic treatment (ITA) against both mites **(single-choice drop-down menu with increments of 5% + ‘Not applicable’ option)**

P.50. **[P50]** Please rank the following factors according to their **clinical relevance when recommending aetiological treatment with ITA against *Dermatophagoides spp.* and *B. tropicalis*** in patients diagnosed with an allergy to both mites.

Rank them from 1 to 11, with 1 being the most relevant and 11 the least relevant

- **[P50_1]** Severity of symptoms
- **[P50_2]** Impact on quality of life
- **[P50_3]** Geographical area of origin
- **[P50_4]** Geographical area of residence
- **[P50_5]** Frequency of travel to hot and humid areas
- **[P50_6]** Occupational activity and/or characteristics
- **[P50_7]** Household characteristics
- **[P50_8]** Leisure activities in hot and humid environments
- **[P50_9]** Clinical pattern of symptoms (time of onset, frequency, association with specific daily routines, cyclical nature, etc.)
- **[P50_10]** Inadequate symptom control (with standard treatment and specific recommendations)
- **[P50_11]** Failure of/response to previous treatment with ITA
- **[P50_12]** Other
  - **P50_OTHERS ______________**

P.51. **[P51]** Please rate these same attributes on a scale of 1 to 10 according to their **clinical relevance when recommending aetiological treatment with ITA versus *Dermatophagoides spp.* and *B. tropicalis*** in patients diagnosed with an allergy to both mites.

Rate from 1 to 10, where 1 is not at all important and 10 is very important

|  | **Not at all important** |  |  |  |  |  |  |  |  | **Very important** |
| --- | --- | --- | --- | --- | --- | --- | --- | --- | --- | --- |
|  | **1** | **2** | **3** | **4** | **5** | **6** | **7** | **8** | **9** | **10** |
| **[P51_1]** Severity of symptoms | □ | □ | □ | □ | □ | □ | □ | □ | □ | □ |
| **[P51_2]** Impact on quality of life | □ | □ | □ | □ | □ | □ | □ | □ | □ | □ |
| **[P51_3]** Geographical area of origin | □ | □ | □ | □ | □ | □ | □ | □ | □ | □ |
| **[P51_4]** Geographical area of residence | □ | □ | □ | □ | □ | □ | □ | □ | □ | □ |
| **[P51_5]** Frequency of travel to hot and humid areas | □ | □ | □ | □ | □ | □ | □ | □ | □ | □ |
| **[P51_6]** Occupational activity and/or characteristics | □ | □ | □ | □ | □ | □ | □ | □ | □ | □ |
| **[P51_7]** Household characteristics | □ | □ | □ | □ | □ | □ | □ | □ | □ | □ |
| **[P51_8]** Recreational activities in warm and humid environments | □ | □ | □ | □ | □ | □ | □ | □ | □ | □ |
| **[P51_9]** Clinical pattern of symptoms (time of onset, frequency, association with specific daily routines, cyclical nature, etc.) | □ | □ | □ | □ | □ | □ | □ | □ | □ | □ |
| **[P51_10]** Poor symptom control (with standard treatment and specific recommendations) | □ | □ | □ | □ | □ | □ | □ | □ | □ | □ |
| **[P51_11]** Failure/response to previous treatment with ITA | □ | □ | □ | □ | □ | □ | □ | □ | □ | □ |
| **[P51_12]** Other | □ | □ | □ | □ | □ | □ | □ | □ | □ | □ |

P.52. **[P52]** Considering your current professional practice as a whole, approximately what percentage of the patients you see on average each month who have **been diagnosed with an allergy to *Dermatophagoides spp.* and *L. destructor* do you recommend etiological treatment with ITA against both mites**?

Please enter your answer (think in terms of patients rather than consultations, and base your answer on a percentage for a month that is representative of the rest of the year). If you do not treat any patients with allergies to Dermatophagoides spp. and B. tropicalis, please select the ‘Not applicable’ option.

________% of patients with allergies to *Dermatophagoides spp*. and *B. tropicalis* for whom you recommend aphidic treatment (ITA) against both mites **(single-choice drop-down menu with 5% increments + ‘Not applicable’ option)**

P.53. **[P53]** Please rank the following factors according to their **clinical relevance when recommending aetiological treatment with ITA against *Dermatophagoides spp. and L. destructor*** in patients diagnosed with an allergy to both mites.

Rank them from 1 to 11, with 1 being the most relevant and 11 the least relevant

- **[P53_1]** Severity of symptoms
- **[P53_2]** Impact on quality of life
- **[P53_3]** Geographical area of origin
- **[P53_4]** Geographical area of residence
- **[P53_5]** Frequency of travel to hot and humid areas
- **[P53_6]** Occupational activity and/or characteristics
- **[P53_7]** Household characteristics
- **[P53_8]** Recreational activities in warm and humid environments
- **[P53_9]** Clinical pattern of symptoms (time of onset, frequency, association with specific daily routines, cyclical nature, etc.)
- **[P53_10]** Lack of symptom control (despite standard treatment and specific recommendations)
- **[P53_11]** Failure of/response to previous treatment with ITA
- **[P53_12]** Other
  - **P53_OTHERS ______________**

P.54. **[P54]** Please rate these same attributes on a scale of 1 to 10 according to their **clinical relevance when recommending aetiological treatment with ITA against *Dermatophagoides spp.* and *L. destructor*** in patients diagnosed with an allergy to both mites.

Rate from 1 to 10, where 1 is not at all important and 10 is very important

|  | **Not at all important** |  |  |  |  |  |  |  |  | **Very important** |
| --- | --- | --- | --- | --- | --- | --- | --- | --- | --- | --- |
|  | **1** | **2** | **3** | **4** | **5** | **6** | **7** | **8** | **9** | **10** |
| **[P54_1]** Severity of symptoms | □ | □ | □ | □ | □ | □ | □ | □ | □ | □ |
| **[P54_2]** Impact on quality of life | □ | □ | □ | □ | □ | □ | □ | □ | □ | □ |
| **[P54_3]** Geographical area of origin | □ | □ | □ | □ | □ | □ | □ | □ | □ | □ |
| **[P54_4]** Geographical area of residence | □ | □ | □ | □ | □ | □ | □ | □ | □ | □ |
| **[P54_5]** Frequency of trips to hot and humid areas | □ | □ | □ | □ | □ | □ | □ | □ | □ | □ |
| **[P54_6]** Occupational activity and/or characteristics | □ | □ | □ | □ | □ | □ | □ | □ | □ | □ |
| **[P54_7]** Household characteristics | □ | □ | □ | □ | □ | □ | □ | □ | □ | □ |
| **[P54_8]** Recreational activities in warm and humid environments | □ | □ | □ | □ | □ | □ | □ | □ | □ | □ |
| **[P54_9]** Clinical pattern of symptoms (time of onset, frequency, association with specific daily routines, cyclical nature, etc.) | □ | □ | □ | □ | □ | □ | □ | □ | □ | □ |
| **[P54_10]** Poor symptom control (with standard treatment and specific recommendations) | □ | □ | □ | □ | □ | □ | □ | □ | □ | □ |
| **[P54_11]** Failure/response to prior treatment with ITA | □ | □ | □ | □ | □ | □ | □ | □ | □ | □ |
| **[P54_12]** Other | □ | □ | □ | □ | □ | □ | □ | □ | □ | □ |

P.55. **[P55]** Do you consider that there is **cross-reactivity between *Dermatophagoides* spp. and *B. tropicalis***?

Please tick one answer only.

- **[1]** Yes🡪 Show P56
- **[2]** No

P.56. **[P56]** Do you consider this to be **relevant** when prescribing aetiological treatment with ITA?
      - **[1]** Yes
      - **[2]** No
P.57. **[P57]**  Please rank the following factors according to their **influence when selecting a causative treatment with ITA against *Dermatophagoides* and *B. tropicalis*** in patients diagnosed with an allergy to both mites.

Treatment-related

Rank from 1 to 7, with 1 being the most important and 7 the least important

- **[P57_1]** Safety
- **[P57_2]** Proven efficacy
- **[P57_3]** Availability of a characterised and standardised extract
- **[P57_4]** Availability of a native extract
- **P57_5]** Availability of a polymerised extract
- **[P57_6]** Availability of an extract containing mixtures at full/optimised doses
- **[P57_7]** Other
  - **P57_OTHERS______________**

Rank from 1 to 9, with 1 being the most important and 9 the least important

P.58. **[P58]**  Please rank the following factors in order of their **influence when selecting a causative treatment for ITA against *Dermatophagoides* and *B. tropicalis*** in patients diagnosed with an allergy to both mites.

Patient-related factors

- **[P58_1]** Sensitisation to different allergenic sources
- **[P58_2]** Sensitisation and confirmed clinical history of allergy to various allergenic sources
- **[P58_3]** Degree of the patient’s exposure to the responsible allergens
- **[P58_4]** Difficulty in avoiding the allergens implicated in the symptoms
- **[P58_5]** Preferences of the patient or responsible family member
- **[P58_6]** Adherence to treatment
- **[P58_7]** Need for other pharmacological treatments (antihistamines, corticosteroids, anti-leukotrienes, reliever inhalers, dual therapy, triple therapy)
- **[P58_8]** Cost per year
- **[P58_9]** Other
  - **P58_OTHERS______________**

P.59. **[P59]** Please rate these same attributes on a scale of 1 to 10 according to their **influence when selecting a causative treatment with ITA versus *Dermatophagoides* and *B. tropicalis*** in patients diagnosed with an allergy to both mites.

Rate from 1 to 10, where 1 is not at all important and 10 is very important

Relating to treatment

|  | **Not at all important** |  |  |  |  |  |  |  |  | **Very important** |
| --- | --- | --- | --- | --- | --- | --- | --- | --- | --- | --- |
|  | **1** | **2** | **3** | **4** | **5** | **6** | **7** | **8** | **9** | **10** |
| **[P59_1]** Safety | □ | □ | □ | □ | □ | □ | □ | □ | □ | □ |
| **[P59_2]** Proven effectiveness | □ | □ | □ | □ | □ | □ | □ | □ | □ | □ |
| **[P59_3]** Availability of a characterised and standardised extract | □ | □ | □ | □ | □ | □ | □ | □ | □ | □ |
| **[P59_4]** Availability of a native extract | □ | □ | □ | □ | □ | □ | □ | □ | □ | □ |
| **[P59_5]** Availability of a polymerised extract | □ | □ | □ | □ | □ | □ | □ | □ | □ | □ |
| **[P59_6]** Availability of an extract containing mixtures at full/optimised doses | □ | □ | □ | □ | □ | □ | □ | □ | □ | □ |
| **[P59_7]** Other | □ | □ | □ | □ | □ | □ | □ | □ | □ | □ |

P.60. **[P60]** Please rate these same attributes on a scale of 1 to 10 according to their **influence when selecting a causative treatment with ITA versus *Dermatophagoides* and *B. tropicalis*** in patients diagnosed with an allergy to both mites.

Rate from 1 to 10, where 1 is not at all important and 10 is very important

Relating to the patient

|  | **Not at all important** |  |  |  |  |  |  |  |  | **Very important** |
| --- | --- | --- | --- | --- | --- | --- | --- | --- | --- | --- |
|  | **1** | **2** | **3** | **4** | **5** | **6** | **7** | **8** | **9** | **10** |
| **[P60_1]** Awareness of different sources of allergens | □ | □ | □ | □ | □ | □ | □ | □ | □ | □ |
| **[P60_2]** Awareness and confirmed medical history of allergy to various allergenic sources | □ | □ | □ | □ | □ | □ | □ | □ | □ | □ |
| **[P60_3]** Degree of the patient’s exposure to the responsible allergens | □ | □ | □ | □ | □ | □ | □ | □ | □ | □ |
| **[P60_4]** Difficulty in avoiding the allergens responsible for the symptoms | □ | □ | □ | □ | □ | □ | □ | □ | □ | □ |
| **[P60_5]** Preferences of the patient or responsible relative | □ | □ | □ | □ | □ | □ | □ | □ | □ | □ |
| **[P60_6]** Adherence to treatment | □ | □ | □ | □ | □ | □ | □ | □ | □ | □ |
| **[P60_7]** Need for other symptomatic treatments (antihistamines, corticosteroids, anti-leukotrienes) | □ | □ | □ | □ | □ | □ | □ | □ | □ | □ |
| **[P60_8]** ITA cost per year | □ | □ | □ | □ | □ | □ | □ | □ | □ | □ |
| **[P60_9]** Other | □ | □ | □ | □ | □ | □ | □ | □ | □ | □ |

P.61. **[P61]** Thinking of your patients diagnosed with an allergy to *Dermatophagoides spp.* and *B. tropicalis*, which **route of administration** do you consider most appropriate to ensure better patient adherence to **aetiological treatment with ITA against *Dermatophagoides spp.* and *B. tropicalis***?

Please select only one answer.

- **[1]** Subcutaneous immunotherapy (SCIT)
- **[2]** Sublingual immunotherapy (SLIT)

P.62. **[P62]** With these patients in mind, do you consider **aetiological treatment with ITA using a mixture of extracts of *Dermatophagoides spp.* and *B. tropicalis***?

Please select only one answer.

- **[1]** Yes
- **[2]** No

P.63. **[P63]** For these patients, would you consider **aetiological treatment with ITA using other heterogeneous mixtures of extracts of *Dermatophagoides spp.* and *B. tropicalis* and other allergenic sources**?

Please select only one answer.

- [**1]** Yes 🡪 Show P64
- **[2]** No

P.64. **[P64]** In that case, which other allergen sources?
      - Please tick ALL answers that apply.
        - **[P64_1]** Pollen
        - **[P64_2]** Animals
        - **[P64_3]** Other mites
        - **[P64_OTHERS]** Other [specify]
P.65. **[P65]** With these patients in mind, **what factors lead you to initiate aetiological treatment with ITA for *Dermatophagoides spp.* and *B. tropicalis*?** How often?

Rate on a scale of 0 to 9, where 0 means ‘never’ and 9 means ‘always’

|  | **Never** |  |  |  |  |  |  |  |  | **Always** |
| --- | --- | --- | --- | --- | --- | --- | --- | --- | --- | --- |
|  | **0** | **1** | **2** | **3** | **4** | **5** | **6** | **7** | **8** | **9** |
| **[P65_1]** At the time of diagnosis | □ | □ | □ | □ | □ | □ | □ | □ | □ | □ |
| **[P65_2]** In the absence of improvement with symptomatic treatment, in accordance with specific recommendations | □ | □ | □ | □ | □ | □ | □ | □ | □ | □ |
| **[P65_3]**. Mild persistent illness | □ | □ | □ | □ | □ | □ | □ | □ | □ | □ |
| **[P65_4]** Moderate persistent illness | □ | □ | □ | □ | □ | □ | □ | □ | □ | □ |
| **[P65_5]** Severe persistent illness | □ | □ | □ | □ | □ | □ | □ | □ | □ | □ |
| **[P65_6]** Patient choice | □ | □ | □ | □ | □ | □ | □ | □ | □ | □ |
| **[P65_7]** Adverse effects of symptomatic treatment | □ | □ | □ | □ | □ | □ | □ | □ | □ | □ |
| **[P65_8]** Other |  |  |  |  |  |  |  |  |  |  |

P.66. **[P66]** Do you use any **clinical guidelines or algorithms when determining the aetiological treatment with ITA versus *Dermatophagoides spp.* and *B. tropicalis*** in patients allergic to both mites?

Please select only one answer.

- **[1]** Yes🡪 Show P67
- **[2]** No

P.67. **[P67]** In that case, which one(s)?
      - Please tick ALL answers that apply
      - **[P67_1]** EAACI
      - **[P67_2]** SEAIC
      - **[P67_3]** AAAAI
      - **[P67_OTHERS]** Others (please specify):_______
P.68. **[P68]** When **prescribing a causative treatment with ITA against *Dermatophagoides spp.* and *B. tropicalis*** in patients allergic to both mites, **what is your main motivation**?

Please tick one answer only.

- **[1]** Impact on the severity of symptoms
- **[2]** Impact on quality of life
- **[3]** Published evidence on the efficacy of the treatment
- **[4]** Preventing the development/progression of respiratory conditions (AA, RA, RCA, DA)
- **[5]** Preventing sensitisation to new allergens
- [**P65_OTROS]** Other
  - **______________**

P.69. **[P69] Does** the **available scientific evidence** **influence your decision** to use aetiological treatment with ITA **against *Dermatophagoides spp.* and *B. tropicalis*** in patients allergic to both mites?

Please tick only one answer for each option.

- [**P69_1] Evidence from clinical trials**
  - **[1]** Yes
  - **[2]** No
- [**P69_2] Real-world evidence**
  - **[1]** Yes
  - **[2]** No

P.70. **[P70]** Do you consider that there is **cross-reactivity between *Dermatophagoides spp.* and *L. destructor***?

Please tick one answer only.

- **[1]** Yes🡪 Show P71
- **[2]** No

P.71. **[P71]** Do you consider this to be relevant when prescribing aetiological treatment with ITA?
      - **[1]** Yes
      - **[2]** No
P.72. **[P72]** Please rank the following factors according to their **influence when selecting a causative treatment with ITA for *Dermatophagoides* and *L. destructor*** in patients diagnosed with an allergy to both mites.

Rank them from 1 to 7, with 1 being the most important and 7 the least important

Treatment-related

- **[P72_1]** Safety
- **[P72_2]** Proven efficacy
- **[P72_3]** Availability of a characterised and standardised extract
- **[P72_4]** Availability of a native extract
- **[P72_5]** Availability of a polymerised extract
- **[P72_6]** Availability of an extract containing mixtures at full/optimised doses
- **[P72_7]** Other
  - **P72_OTHERS______________**

P.73. **[P73]** Please rank the following factors in order of their **influence when selecting a causative treatment for ITA against *Dermatophagoides* and *L. destructor*** in patients diagnosed with an allergy to both mites.

Rank them from 1 to 8, with 1 being the most important and 8 the least important

Patient-related factors

- **[P73_1]** Sensitisation to different allergenic sources
- **[P73_2]** Sensitisation and confirmed clinical history of allergy to various allergenic sources
- **[P73_3]** Degree of the patient’s exposure to the responsible allergens
- **[P73_4]** Difficulty in avoiding the allergens implicated in the symptoms
- **[P73_5]** Preferences of the patient or responsible family member
- **[P73_6]** Adherence to treatment
- **[P73_7]** Need for other pharmacological treatments (antihistamines, corticosteroids, anti-leukotrienes, reliever inhalers, dual therapy, triple therapy)
- **[P73_8]** Cost per year
- **[P73_9]** Other
  - **P73_OTHERS______________**

P.74. **[P74]** Please rate these same attributes on a scale of 1 to 10 according to their **influence when selecting a causative treatment with ITA versus *Dermatophagoides* and *L. destructor*** in patients diagnosed with an allergy to both mites.

Rate from 1 to 10, where 1 is not at all important and 10 is very important

Relating to treatment

|  | **Not at all important** |  |  |  |  |  |  |  |  | **Very important** |
| --- | --- | --- | --- | --- | --- | --- | --- | --- | --- | --- |
|  | **1** | **2** | **3** | **4** | **5** | **6** | **7** | **8** | **9** | **10** |
| **[P74_1]** Safety | □ | □ | □ | □ | □ | □ | □ | □ | □ | □ |
| **[P74_2]** Proven effectiveness | □ | □ | □ | □ | □ | □ | □ | □ | □ | □ |
| **[P74_3]** Availability of a characterised and standardised extract | □ | □ | □ | □ | □ | □ | □ | □ | □ | □ |
| **[P74_4]** Availability of a native extract | □ | □ | □ | □ | □ | □ | □ | □ | □ | □ |
| **[P74_5]** Availability of a polymerised extract | □ | □ | □ | □ | □ | □ | □ | □ | □ | □ |
| **[P74_6]** Availability of an extract containing mixtures at full/optimised doses | □ | □ | □ | □ | □ | □ | □ | □ | □ | □ |
| **[P74_7]** Other | □ | □ | □ | □ | □ | □ | □ | □ | □ | □ |

P.75. **[P75]** Please rate these same attributes on a scale of 1 to 10 according to their **influence when selecting a causative treatment using ITA versus *Dermatophagoides* and *L. destructor*** in patients diagnosed with an allergy to both mites.

Rate on a scale of 1 to 10, where 1 is not at all important and 10 is very important

Relating to the patient

|  | **Not at all important** |  |  |  |  |  |  |  |  | **Very important** |
| --- | --- | --- | --- | --- | --- | --- | --- | --- | --- | --- |
|  | **1** | **2** | **3** | **4** | **5** | **6** | **7** | **8** | **9** | **10** |
| **[P75_1]** Awareness of different sources of allergens | □ | □ | □ | □ | □ | □ | □ | □ | □ | □ |
| **[P75_2]** Awareness and confirmed medical history of allergy to various allergenic sources | □ | □ | □ | □ | □ | □ | □ | □ | □ | □ |
| **[P75_3]** Degree of the patient’s exposure to the responsible allergens | □ | □ | □ | □ | □ | □ | □ | □ | □ | □ |
| **[P75_4]** Difficulty in avoiding the allergens responsible for the symptoms | □ | □ | □ | □ | □ | □ | □ | □ | □ | □ |
| **[P75_5]** Preferences of the patient or responsible relative | □ | □ | □ | □ | □ | □ | □ | □ | □ | □ |
| **[P75_6]** Adherence to treatment | □ | □ | □ | □ | □ | □ | □ | □ | □ | □ |
| **[P75_7]** Need for other pharmacological treatments (antihistamines, corticosteroids, anti-leukotrienes, reliever inhalers, dual therapy, triple therapy) | □ | □ | □ | □ | □ | □ | □ | □ | □ | □ |
| **[P75_8]** Cost per year | □ | □ | □ | □ | □ | □ | □ | □ | □ | □ |
| **[P75_9]** Other | □ | □ | □ | □ | □ | □ | □ | □ | □ | □ |

P.76. **[P76]** Thinking of your patients with a diagnosed allergy to *Dermatophagoides spp.* and *L. destructor*, which **route of administration** do you consider most appropriate to ensure **better patient adherence to aetiological treatment with ITA against *Dermatophagoides spp.* and *L. destructor***?

Please select only one answer.

- **[1]** Subcutaneous immunotherapy (SCIT)
- **[2]** Sublingual immunotherapy (SLIT)

P.77. **[P77]** With these patients in mind, do you consider **aetiological treatment with ITA using a mixture of extracts of *Dermatophagoides spp.* and *L. destructor***?

Please select only one answer.

- **[1]** Yes
- **[2]** No

P.78. **[P78]** With these patients in mind, do you consider **aetiological treatment with ITA using other heterogeneous mixtures of extracts from *Dermatophagoides spp.* and *L. destructor* and other allergenic sources**?

Please select only one answer.

- [**1]** Yes 🡪 Show P79
- **[2]** No

P.79. **[P79]** In that case, which other allergen sources?
      - Please tick ALL answers that apply.
      - **[P79_1]** Pollen
      - **[P79_2]** Animals
      - **[P79_3]** Other dust mites
      - **[P79_OTHERS]** Others
P.80. **[P80]** With these patients in mind, **what factors lead you to initiate aetiological treatment with ITA for *Dermatophagoides spp.* and *L. destructor*?** How often?

Rate on a scale of 0 to 9, where 0 means ‘never’ and 9 means ‘always’

|  | **Never** |  |  |  |  |  |  |  |  | **Always** |
| --- | --- | --- | --- | --- | --- | --- | --- | --- | --- | --- |
|  | **0** | **1** | **2** | **3** | **4** | **5** | **6** | **7** | **8** | **9** |
| **[P80_1]** At the time of diagnosis | □ | □ | □ | □ | □ | □ | □ | □ | □ | □ |
| **[P80_2]** In the absence of improvement with symptomatic treatment, in accordance with specific recommendations | □ | □ | □ | □ | □ | □ | □ | □ | □ | □ |
| **[P80_3]**. Mild persistent illness | □ | □ | □ | □ | □ | □ | □ | □ | □ | □ |
| **[P80_4]** Moderate persistent illness | □ | □ | □ | □ | □ | □ | □ | □ | □ | □ |
| **[P80_5]** Severe chronic illness | □ | □ | □ | □ | □ | □ | □ | □ | □ | □ |
| **[P80_6]** Patient choice | □ | □ | □ | □ | □ | □ | □ | □ | □ | □ |
| **[P80_7]** Adverse effects of symptomatic treatment | □ | □ | □ | □ | □ | □ | □ | □ | □ | □ |
| **[P80_8]** Other |  |  |  |  |  |  |  |  |  |  |

P.81. **[P81]** Do you use any **clinical guidelines or algorithms when determining the aetiological treatment with ITA versus *Dermatophagoides spp.* and *L. destructor*** in patients allergic to both mites?

Please select only one answer.

- **[1]** Yes🡪 Show P82
- **[2]** No

P.82. **[P82]** In that case, which one(s)?
      - Please tick ALL the answers that apply.
        - **[P82_1]** EAACI
        - **[P82_2]** SEAIC
        - **[P82_3]** AAAAI
        - **[P82_OTHERS]** Others
P.83. **[P83]** When **prescribing a causative treatment with ITA against *Dermatophagoides spp.* and *L. destructor*** in patients allergic to both mites, **what is your main motivation**?

Please select only one answer.

- **[1]** Impact on the severity of symptoms
- **[2]** Impact on quality of life
- **[3]** Published evidence on the efficacy of the treatment
- **[5]** Preventing the development/progression of respiratory conditions (AA, RA, RCA, DA)
- **[5]** Preventing sensitisation to new allergens
- **[6]** Other
  - **[P83_OTHERS] Specify others: ______________**

P.84. **[P84] Does** the **available scientific evidence** **influence your decision** to use aetiological treatment with ITA **against *Dermatophagoides spp.* and *L. destructor*** in patients allergic to both mites?

Please tick only one answer for each option.

- **[P84_1] Evidence from clinical trials**
  - **[1]** Yes
  - **[2]** No
- **[P84_2] Real-world evidence**
  - **[1]** Yes
  - **[2]** No

Barriers to treatment with ITA

P.85. **[P85]** Please rank the following factors according to **the extent to which they hinder the determination of aetiological treatment with ITA against *Dermatophagoides spp.* and *B. tropicalis*** in patients diagnosed with an allergy to both mites.

Rank them from 1 to 7, with 1 being the most important and 7 the least important

Relating to treatment

- **[P85_1]** Strength of the scientific evidence
- **[P85_2]** Clear clinical guidelines/protocols
- **[P85_3]** Cross-reactivity between species
- **[P85_4]** Lack of consensus on biomarkers to measure treatment efficacy
- **[P85_5]** Availability of polymerised, well-characterised and standardised extracts that guarantee the stability, safety and efficacy of the mixtures
- **[P85_6]** Adverse events
- **P85_7]** Other
  - **P85_OTHERS______________**

P.86. **[P86]** Please rank the following factors according to **the extent to which they hinder the determination of aetiological treatment with ITA versus *Dermatophagoides spp.* and *B. tropicalis*** in patients diagnosed with an allergy to both mites.

Rank from 1 to 5, with 1 being the most important and 5 the least important

Patient-related factors

- **[P86_1]** Cost of treatment
- **[P86_2]** Patient consent
- **[P86_3]** Patient adherence
- **[P86_4]** Use of administrative resources in relation to symptomatic treatment (approval, consultation time, etc.)
- [**P86_5]** Other
  - **P86_OTHERS______________**

P.87. **[P87]** Please rate these same attributes on a scale of 1 to 10 according to **the extent to which they hinder the determination of aetiological treatment with ITA versus *Dermatophagoides spp.* and *B. tropicalis*** in patients diagnosed with an allergy to both mites.

Rate from 1 to 10, where 1 is not at all important and 10 is very important

Relating to treatment

|  | **Not at all important** |  |  |  |  |  |  |  |  |  |
| --- | --- | --- | --- | --- | --- | --- | --- | --- | --- | --- |
|  | **1** | **2** | **3** | **4** | **5** | **6** | **7** | **8** | **9** | **10** |
| **[P87_1]** Strength of the scientific evidence | □ | □ | □ | □ | □ | □ | □ | □ | □ | □ |
| **[P87_2]** Clear clinical guidelines/protocols | □ | □ | □ | □ | □ | □ | □ | □ | □ | □ |
| **[P87_3]** Cross-reactivity between species | □ | □ | □ | □ | □ | □ | □ | □ | □ | □ |
| **[P87_4]** Lack of consensus on biomarkers to measure treatment efficacy | □ | □ | □ | □ | □ | □ | □ | □ | □ | □ |
| **[P87_5]** To have access to polymerised extracts that are well characterised and standardised, ensuring the stability, safety and efficacy of the mixtures | □ | □ | □ | □ | □ | □ | □ | □ | □ | □ |
| **[P87_6]**  Adverse events | □ | □ | □ | □ | □ | □ | □ | □ | □ | □ |
| **[P87_7]** Other | □ | □ | □ | □ | □ | □ | □ | □ | □ | □ |

P.88. **[P88]** Please rate these same attributes on a scale of 1 to 10 according to **the extent to which they hinder the determination of aetiological treatment with ITA versus *Dermatophagoides spp.* and *B. tropicalis*** in patients diagnosed with an allergy to both mites.

Rate from 1 to 10, where 1 is not at all important and 10 is very important

Relating to the patient

|  | **Not at all important** |  |  |  |  |  |  |  |  | **Very significant** |
| --- | --- | --- | --- | --- | --- | --- | --- | --- | --- | --- |
|  | **1** | **2** | **3** | **4** | **5** | **6** | **7** | **8** | **9** | **10** |
| **[P88_1]** Cost of treatment | □ | □ | □ | □ | □ | □ | □ | □ | □ | □ |
| **[P88_2]** Patient consent | □ | □ | □ | □ | □ | □ | □ | □ | □ | □ |
| **[P88_3]** Patient adherence | □ | □ | □ | □ | □ | □ | □ | □ | □ | □ |
| **[P88_4]** Use of administrative resources compared with symptomatic treatment (approval, consultation time, etc.) |  |  |  |  |  |  |  |  |  |  |
| **[P88_5]** Other | □ | □ | □ | □ | □ | □ | □ | □ | □ | □ |

P.89. **[P89]** Please rank the following factors according to **the extent to which they hinder the determination of aetiological treatment with ITA versus *Dermatophagoides spp.* and *L. destructor*** in patients diagnosed with an allergy to both mites.

Rank them from 1 to 6, with 1 being the most important and 6 the least important

Relating to treatment

- **[P89_1]** Strength of the scientific evidence
- **[P89_2]** Clear clinical guidelines/protocols
- **[P89_3]** Cross-reactivity between species
- **[P89_4]** Lack of consensus on biomarkers to measure treatment efficacy
- **[P89_5]** Availability of polymerised, well-characterised and standardised extracts that guarantee the stability, safety and efficacy of the mixtures
- **[P89_6]** Adverse events
- **[P89_7]** Other
  - **P89_OTHERS______________**

P.90. **[** **P9 0]** Please rank the following factors according to **the extent to which they hinder the determination of aetiological treatment with ITA versus *Dermatophagoides spp.* and *L. destructor*** in patients diagnosed with an allergy to both mites.

Rank them from 1 to 5, with 1 being the most important and 5 the least important

Patient-related factors

- **[P90_1]** Cost of treatment
- **[P90_2]** Patient consent
- **[P90_3]** Patient adherence
- **[P90_4]** Use of administrative resources compared with symptomatic treatment (approval, consultation time, etc.)
- **[P90_5]** Other
  - **P90_OTHERS______________**

P.91. **[P91]** Please rate these same attributes on a scale of 1 to 10 according to **the extent to which they hinder the determination of aetiological treatment with ITA versus *Dermatophagoides spp.* and *L. destructor*** in patients diagnosed with an allergy to both mites.

Rate from 1 to 10, where 1 is not at all important and 10 is very important

Relating to treatment

|  | **Not at all important** |  |  |  |  |  |  |  |  |  |
| --- | --- | --- | --- | --- | --- | --- | --- | --- | --- | --- |
|  | **1** | **2** | **3** | **4** | **5** | **6** | **7** | **8** | **9** | **10** |
| **[P91_1]** Strength of the scientific evidence | □ | □ | □ | □ | □ | □ | □ | □ | □ | □ |
| **[P91_2]** Clear clinical guidelines/protocols | □ | □ | □ | □ | □ | □ | □ | □ | □ | □ |
| **[P91_3]** Cross-reactivity between species | □ | □ | □ | □ | □ | □ | □ | □ | □ | □ |
| **[P91_4]** Lack of consensus on biomarkers to measure treatment efficacy | □ | □ | □ | □ | □ | □ | □ | □ | □ | □ |
| **[P91_5]** To have access to polymerised extracts that are well characterised and standardised, ensuring the stability, safety and efficacy of the mixtures | □ | □ | □ | □ | □ | □ | □ | □ | □ | □ |
| **[P91_6]**  Adverse events | □ | □ | □ | □ | □ | □ | □ | □ | □ | □ |
| **[P91_7]** Other | □ | □ | □ | □ | □ | □ | □ | □ | □ | □ |

P.92. **[P92]** Please rate these same attributes on a scale of 1 to 10 according to **the extent to which they hinder the determination of aetiological treatment with ITA versus *Dermatophagoides spp.* and *L. destructor*** in patients diagnosed with an allergy to both mites.

Rate from 1 to 10, where 1 is not at all important and 10 is very important

Relating to the patient

|  | **Not at all important** |  |  |  |  |  |  |  |  | **Very significant** |
| --- | --- | --- | --- | --- | --- | --- | --- | --- | --- | --- |
|  | **1** | **2** | **3** | **4** | **5** | **6** | **7** | **8** | **9** | **10** |
| **[P92_1]** Cost of treatment | □ | □ | □ | □ | □ | □ | □ | □ | □ | □ |
| **[P92_2]** Patient consent | □ | □ | □ | □ | □ | □ | □ | □ | □ | □ |
| **[P92_3]** Patient adherence | □ | □ | □ | □ | □ | □ | □ | □ | □ | □ |
| **[P92_4]** Use of administrative resources compared with symptomatic treatment (approval, consultation time, etc.) |  |  |  |  |  |  |  |  |  |  |
| **[P92_5]** Other | □ | □ | □ | □ | □ | □ | □ | □ | □ | □ |

Opportunities in treatment with ITA

P.93. **[P93]** Please rank the following factors according to their **potential importance in facilitating aetiological treatment with ITA against *B. tropicalis*** in patients diagnosed with an allergy to both mites.

Rank them from 1 to 6, with 1 being the most important and 6 the least important

- **[P93_1]** Availability of polymerised, well-characterised and standardised extracts that guarantee the stability, safety and efficacy of the mixtures
- **[P93_2]** Optimisation of the dosage regimen (extracts administered twice a year)
- **[P93_3]** To have standardised guidelines/protocols tailored to the patient and their environment
- **[P93_4]** Personalisation of treatment
- **[P93_5]** Greater clinical efficacy of treatment
- [**P93_6]** Other
  - **P93_OTHERS______________**

P.94. **[P94]** Please rate these same attributes on a scale of 1 to 10 according to their **potential importance in facilitating aetiological treatment with ITA versus *B. tropicalis*** in patients diagnosed with an allergy to both mites.

Rate from 1 to 10, where 1 is not at all important and 10 is very important

|  | **Not at all important** |  |  |  |  |  |  |  |  | **Very important** |
| --- | --- | --- | --- | --- | --- | --- | --- | --- | --- | --- |
|  | **1** | **2** | **3** | **4** | **5** | **6** | **7** | **8** | **9** | **10** |
| **[P94_1]** To have access to polymerised, well-characterised and standardised extracts that guarantee the stability, safety and efficacy of the mixtures | □ | □ | □ | □ | □ | □ | □ | □ | □ | □ |
| **[P94_2]** Optimisation of dosage (extracts administered twice a year) | □ | □ | □ | □ | □ | □ | □ | □ | □ | □ |
| **[P94_3]** To have standardised guidelines/protocols tailored to the patient and their environment | □ | □ | □ | □ | □ | □ | □ | □ | □ | □ |
| **[P94_4]**  Personalisation of treatment | □ | □ | □ | □ | □ | □ | □ | □ | □ | □ |
| **[P94_5]** Greater clinical efficacy of the treatment | □ | □ | □ | □ | □ | □ | □ | □ | □ | □ |
| **[P94_6]** Other | □ | □ | □ | □ | □ | □ | □ | □ | □ | □ |

P.95. **[P95]** Please rank the following factors according to their **potential importance in facilitating aetiological treatment with ITA versus *L. destructor*** in patients diagnosed with an allergy to both mites.

Rank them from 1 to 6, with 1 being the most important and 6 the least important

- **[P95_1]** Availability of polymerised, well-characterised and standardised extracts that guarantee the stability, safety and efficacy of the mixtures
- **[P95_2]** Optimisation of the dosage regimen (extracts administered twice a year)
- **[P95_3]** Availability of standardised guidelines/protocols tailored to the patient and their environment
- **[P95_4]** Personalisation of treatment
- **[P95_5]** Greater clinical efficacy of treatment
- **[P95_6]** Other
  - **P95_OTHERS______________**

P.96. **[P96]** Please rate these same attributes on a scale of 1 to 10 according to their **potential importance in facilitating aetiological treatment with ITA versus *L. destructor*** in patients diagnosed with an allergy to both mites.

Rate from 1 to 10, where 1 is not at all important and 10 is very important

|  | **Not at all important** |  |  |  |  |  |  |  |  | **Very important** |
| --- | --- | --- | --- | --- | --- | --- | --- | --- | --- | --- |
|  | **1** | **2** | **3** | **4** | **5** | **6** | **7** | **8** | **9** | **10** |
| **[P96_1]** To have access to polymerised, well-characterised and standardised extracts that guarantee the stability, safety and efficacy of the mixtures | □ | □ | □ | □ | □ | □ | □ | □ | □ | □ |
| **[P96_2]** Optimisation of dosage (extracts administered twice a year) | □ | □ | □ | □ | □ | □ | □ | □ | □ | □ |
| **[P96_3]** To have standardised guidelines/protocols tailored to the patient and their environment | □ | □ | □ | □ | □ | □ | □ | □ | □ | □ |
| **[P96_4]** Personalisation of treatment | □ | □ | □ | □ | □ | □ | □ | □ | □ | □ |
| **[P96_5]** Greater clinical efficacy of the treatment | □ | □ | □ | □ | □ | □ | □ | □ | □ | □ |
| **[P96_6]** Other | □ | □ | □ | □ | □ | □ | □ | □ | □ | □ |

Thank you very much for your participation and your interest in being part of the **MITE-WISE (*Mite Immunotherapy Expert Wisdom & Evidence*)** project**.**

**Supplementary 2:**

**Semi-structured Discussion Guide for Qualitative Expert Interviews**

**Purpose of the qualitative interviews**

The qualitative component of the MITE-WISE project consisted of semi-structured online interviews with eight allergists from Spain and Portugal with longstanding experience in mite allergy and allergen immunotherapy (AIT).

The purpose of these interviews was to provide additional context for the findings of the preceding quantitative questionnaire and to explore how the survey results reflected clinical practice across different geographical settings.

The discussions focused on regional patterns of mite sensitization, diagnostic assessment of *Blomia tropicalis* and *Lepidoglyphus destructor*, factors influencing diagnostic and therapeutic decision-making, barriers to diagnosis and treatment, and areas where additional evidence or clinical guidance may be needed.

The following topics and questions were used to guide the discussions.

**1. Introduction and clinical context**

Experts were initially invited to briefly describe their professional background and clinical setting.

Topics included:

- geographical region and healthcare setting;
- experience in the management of patients with mite allergy;
- experience with patients sensitized or allergic to *Dermatophagoides* spp., *B. tropicalis*, and/or *L. destructor*;
- specific characteristics of mite allergy encountered in their geographical area.

Experts were informed that the discussion was intended to capture their individual clinical experience and their perception of clinical practice in their region.

**2. Regional patterns of mite sensitization and allergy**

Experts were asked to discuss the occurrence and perceived clinical relevance of *B. tropicalis* and *L. destructor* in patients with *Dermatophagoides* spp. allergy.

**Discussion topics**

- How frequently are patients with *Dermatophagoides* spp. and *B. tropicalis* encountered in your clinical setting?
- How frequently are patients with *Dermatophagoides* spp. and *L. destructor* encountered?
- Do the quantitative survey findings broadly reflect your clinical experience?
- Are these sensitization/allergy patterns similar or different in your geographical area?
- What factors may contribute to the patterns observed in your region?
- Have you perceived changes over time in the clinical relevance of *B. tropicalis* or *L. destructor*?
- Are there particular geographical or environmental characteristics that you consider relevant when interpreting mite sensitization patterns?

**Additional topics for exploration, where relevant**

- geographical area of residence or origin;
- temperature and humidity;
- local environmental exposure;
- population mobility;
- regional variation in mite exposure;
- possible changes in sensitization profiles over time.

**3. Diagnostic assessment of *B. tropicalis* and *L. destructor***

Experts were invited to discuss how sensitization to *B. tropicalis* and *L. destructor* is investigated in patients with *Dermatophagoides* spp. allergy.

**Discussion topics**

- In patients with *Dermatophagoides* spp. allergy, under what circumstances do you investigate sensitization to *B. tropicalis*?
- Under what circumstances do you investigate sensitization to *L. destructor*?
- Which diagnostic procedures are most useful in your clinical practice?
- How do you interpret the difference observed between testing for *B. tropicalis* and testing for *L. destructor*?
- Does this pattern reflect diagnostic practice in your region?
- What factors influence the decision to include these mites in the diagnostic work-up?
- Are there circumstances in which testing is performed selectively rather than routinely?

**Factors influencing diagnostic testing**

Experts were invited to discuss the relevance of factors identified in the questionnaire, including:

- the patient's geographical area of residence or origin;
- environmental conditions such as temperature and humidity;
- previous AIT failure;
- insufficient symptom control despite standard management.

Experts were also asked to discuss whether other clinical or environmental factors influenced their diagnostic decisions.

**4. Barriers to diagnostic characterization**

**Discussion topics**

- Do these barriers reflect your experience?
- Which barriers are most relevant in your clinical setting?
- How does the availability or absence of regional sensitization data influence diagnostic decisions?
- Would updated regional environmental or acarological information facilitate diagnostic assessment?
- Are there additional barriers that influence whether *B. tropicalis* or *L. destructor* are investigated?

Where relevant, experts were also invited to discuss the role of:

- skin prick testing;
- in vitro testing;
- complete allergen extracts;
- component-resolved diagnostics;
- clinical history;
- specific clinical suspicion.

**5. Geographical variation in diagnostic practice**

**Discussion topics**

- To what extent does geographical context influence diagnostic practice in your region?
- Are *B. tropicalis* and *L. destructor* routinely considered in mite allergy assessment in your setting?
- Are there differences between coastal, humid, inland, or other geographical environments?
- Which environmental or clinical characteristics appear most relevant when determining whether additional mite species should be investigated?
- How could diagnostic approaches be better adapted to local patterns of allergen exposure and sensitization?

**6. Allergen immunotherapy decision-making**

Experts were subsequently asked to discuss AIT use in patients considered to have clinically relevant sensitization to *Dermatophagoides* spp. together with *B. tropicalis* or *L. destructor*.

**Discussion topics**

- How do you decide whether to prescribe AIT in patients with clinically relevant sensitization to *Dermatophagoides* spp. and *B. tropicalis*?
- How do you approach patients with clinically relevant sensitization to *Dermatophagoides* spp. and *L. destructor*?
- Do the high reported AIT prescription rates observed in the survey reflect your clinical experience?
- What clinical factors are most important when deciding whether to recommend AIT?
- Does the approach differ depending on the mite species involved?
- What factors influence the choice of route of administration?
- What factors influence the allergen composition selected for AIT?

**7. Clinical determinants of AIT prescription**

**Discussion topics**

- Which clinical characteristics are most important when considering AIT in patients sensitized to multiple mite species?
- How do symptom severity and symptom control influence your decision?
- How important is the expected impact on patient quality of life?
- How do you establish whether sensitization to an individual mite species is clinically relevant when selecting AIT?

**8. Use of allergen extract mixtures**

**Discussion topics**

- In patients with clinically relevant sensitization to more than one mite species, how do you determine the allergen composition of AIT?
- What factors influence the decision to use allergen extract mixtures?
- How does geographical exposure influence treatment selection?
- What clinical considerations are important when selecting allergens for inclusion in AIT?
- What uncertainties or evidence gaps remain regarding the use of allergen mixtures in polysensitized patients?

**9. Barriers to AIT implementation**

**Discussion topics**

- Do these barriers reflect your clinical experience?
- Which evidence gaps most influence therapeutic decision-making?
- Would additional clinical guidance facilitate treatment selection?
- Are there specific uncertainties regarding the use of AIT in patients sensitized to multiple mite species?
- What additional evidence would be most useful for clinical practice?

**10. Unmet needs and opportunities for improvement**

**Discussion topics**

- What are the main opportunities to improve the diagnosis of patients with clinically relevant sensitization to *B. tropicalis* or *L. destructor*?
- What could facilitate more appropriate diagnostic characterization?
- How could regional differences in mite exposure and sensitization be better incorporated into clinical decision-making?
- What additional clinical evidence or guidance is needed?
- What role could updated acarological or exposomic maps play?
- What role could advanced diagnostic approaches play in selected patients?
- What areas should be prioritized for future research?
- What recommendations would you make to improve the overall diagnostic and therapeutic management of these patients in your region?

**Closing**

Experts were thanked for their participation and for providing their clinical and regional perspective on the quantitative findings of the MITE-WISE project.

Their contributions were used to provide contextual interpretation of the questionnaire results and to identify region-specific factors, diagnostic and therapeutic barriers, and areas for future clinical and research development.
